# Mammalian-brain-inspired neuromorphic motion-cognition nerve achieves cross-modal perceptual enhancement

**DOI:** 10.1038/s41467-023-36935-w

**Published:** 2023-03-11

**Authors:** Chengpeng Jiang, Jiaqi Liu, Yao Ni, Shangda Qu, Lu Liu, Yue Li, Lu Yang, Wentao Xu

**Affiliations:** 1 Institute of Photoelectronic Thin Film Devices and Technology, Key Laboratory of Photoelectronic Thin Film Devices and Technology of Tianjin, College of Electronic Information and Optical Engineering, Engineering Research Center of Thin Film Photoelectronic Technology of Ministry of Education, School of Materials Science and Engineering, Smart Sensing Interdisciplinary Science Center, Nankai University, Tianjin, 300350 China; 2 Shenzhen Research Institute of Nankai University, Shenzhen, 518000 China; 3 Research Center for Intelligent Sensing, Zhejiang Lab, Hangzhou, 311100 China

**Keywords:** Electronic devices, Electronic devices, Electronic properties and materials

## Abstract

Perceptual enhancement of neural and behavioral response due to combinations of multisensory stimuli are found in many animal species across different sensory modalities. By mimicking the multisensory integration of ocular-vestibular cues for enhanced spatial perception in macaques, a bioinspired motion-cognition nerve based on a flexible multisensory neuromorphic device is demonstrated. A fast, scalable and solution-processed fabrication strategy is developed to prepare a nanoparticle-doped two-dimensional (2D)-nanoflake thin film, exhibiting superior electrostatic gating capability and charge-carrier mobility. The multi-input neuromorphic device fabricated using this thin film shows history-dependent plasticity, stable linear modulation, and spatiotemporal integration capability. These characteristics ensure parallel, efficient processing of bimodal motion signals encoded as spikes and assigned with different perceptual weights. Motion-cognition function is realized by classifying the motion types using mean firing rates of encoded spikes and postsynaptic current of the device. Demonstrations of recognition of human activity types and drone flight modes reveal that the motion-cognition performance match the bio-plausible principles of perceptual enhancement by multisensory integration. Our system can be potentially applied in sensory robotics and smart wearables.

## Abstract (web summary)

Inspired by the multisensory cue integration in macaque’s brain for spatial perception, the authors develop a neuromorphic motion-cognition nerve that achieves cross-modal perceptual enhancement for robotics and wearable applications.

## Introduction

Multisensory integration is a process that combines inputs across different sensory modalities. It is essential in achieving high-level perception and cognition in many animal species^1–3^. Combinations of sensory cues benefit the perceptual and cognitive capabilities to fulfill decision-making, memory, and learning tasks, which are implemented in plastic networks of neurons and synapses by parallel, efficient, and event-driven sensory processing of spatiotemporal correlated neural spikes^4,5^. Behavioral and psychological experiments on mammals indicate that combining multiple cues across sensory modalities effectively improves perceptual performance, including neural and behavioral response^6–8^. These observations suggest that perceptual enhancement due to multisensory integration may provide guidelines for implementing biological principles in neuromorphic electronics^9–14^.

Recent attempts at the neuromorphic multisensory system have focused on the fusion of sensory (mainly tactile, visual, and auditory) and memory functions to realize associative learning, memory retention, and pattern recognition (mostly involving artificial neural networks) by using artificial synapses, artificial neurons, or artificial sensory nerves^15–18^. However, the fundamental principles of spatiotemporal processing, perceptual enhancement, inverse effectiveness, and perceptual weighting in multisensory integration have not been sufficiently investigated and compared with biological rules. Neuroscience studies show that some mammals can optimally integrate ocular and vestibular information to solve spatial cognition tasks^6,19,20^, and achieve superior behavioral responses (e.g., accuracy and reaction time) under bimodal sensory stimuli than under unimodal sensory stimuli^21^. Nevertheless, motion or spatial perception that combines the usage of cross-modal sensory inputs with cognitive functions has been little investigated in neuromorphic hardware. It is expected that the simultaneous realization of neuronal functions, cognitive intelligence, and multisensory enhancement in a neuromorphic motion perception system would be a giant leap in its development towards applications in advanced robotics^22^, wearable electronics^23^, and smart interface^24^, wherein energy-efficient sensing and real-time recognition are highly desirable.

Here, we report a bioinspired motion-cognition nerve that uses a flexible multisensory neuromorphic device to emulate the integration of different sensory cues. A facile and scalable fabrication method is developed to prepare a semiconductor thin film of nanoparticle-doped two-dimensional (2D)-nanoflakes. Benefited from the charge-trapping effect of the 2D material and the solution-processing nature of the fabrication protocol, a high-performance flexible neuromorphic device is then developed, showing history-dependent plasticity, stable linear modulation, and spatiotemporal integration capability. Bimodal motion signals of acceleration and angular speed, encoded as spatiotemporal spike trains and assigned with different perceptual weights (weight of sensory cue), are sent to the dual-gate synaptic transistor, which enables parallel and event-based processing of multisensory information. Utilizing hardware outputs of the synaptic electronics, this system is capable of fulfilling motion recognition tasks, including human activity recognition and drone flight-mode recognition. Moreover, multisensory integration of other types of sensory cues (such as visual-vestibular integration and vibrotactile-vestibular integration) can also be realized in our system. The recognition accuracy of our system is higher under bimodal sensory conditions (two types of sensory inputs are integrated) than under unimodal sensory conditions (each type of sensory input is segregated) and exhibits an inverse effectiveness effect, and thereby its motion-cognition functions replicate the biological principles of perceptual enhancement through multisensory integration.

## Results

### Bioinspired neuromorphic motion-cognition nerve

**Fig. 1:**
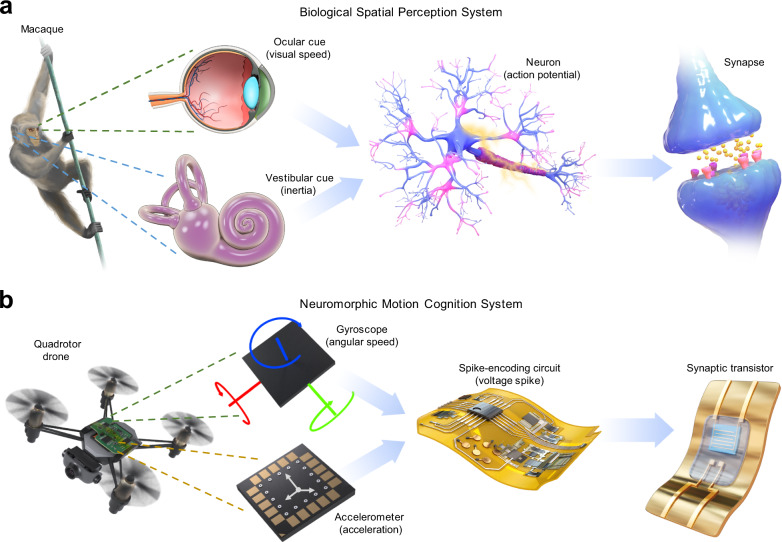
Bioinspired neuromorphic motion-cognition nerve in comparison with an ocular-vestibular cross-modal sensory nerve of macaques. **a** Inertia and visual-speed signals acquired by the vestibular and retina are transmitted as spike trains with spatiotemporal patterns among neurons and synapses, and finally conveyed to head-direction cells and angular-head-velocity cells located in various cortical and subcortical regions of the brain, achieving high-level functions of motion and spatial cognition with multisensory enhancement. **b** Bioinspired neuromorphic motion-cognition nerve built from an inertial measurement unit (IMU) sensor, flexible spike-encoding circuit, and flexible multisensory synaptic transistor. Acceleration and angular speed signals detected by the accelerometer and gyroscope in the IMU sensor were encoded as two spatiotemporal spike trains, then sent to the two gates of the synaptic transistor. Motion-cognition was achieved in a continuous, neuromorphic, event-based manner using hardware outputs.

Our bioinspired neuromorphic motion-cognition nerve is inspired by the cross-modal integration of sensory cues for motion and spatial perception. In macaques, self-motion in the environment invokes inertial stimuli in the inner-ear vestibular, and visual stimuli in the retina (Fig. 1a)^6,25^. The vestibular-inertia and visual-speed information are converted to spike trains carrying different spatiotemporal patterns and then processed in the networks of neurons and synapses through the process of sensory perception and cognition^26^. The integration of information from two different sensory modalities results in neural and behavioral response enhancement regarding motion and spatial perception.

In our system, motion signals of acceleration and angular speed are obtained by an accelerometer and a gyroscope, respectively, of an inertial measurement unit (IMU) sensor (Fig. 1b), in manners analogous to the ocular-vestibular system. The bimodal motion signals corresponding to two sensory modalities (acceleration and rotation) are first converted to two spatiotemporal spike trains by a microcontroller-based flexible spike-encoding circuit using the spike-rate coding method (Fig. S1)^19,27,28^, and then these two spikes trains are transmitted to the dual gates of a flexible synaptic transistor by assigning perceptual weights to different sensory modalities. The temporal correlation and temporal patterns of the two spike trains affect the synaptic potentiation behaviors of the synaptic transistor, and consequently affect the device output (postsynaptic current). In this way, the device realizes a multisensory integration function. Recognition of motion types is implemented by averaging the presynaptic firing rates of the encoded spikes and reading the postsynaptic current of the device in an event-based manner, and thus the neuromorphic cognitive perception of motion information across different sensory modalities is achieved.

### Flexible multisensory synaptic transistor

**Fig. 2:**
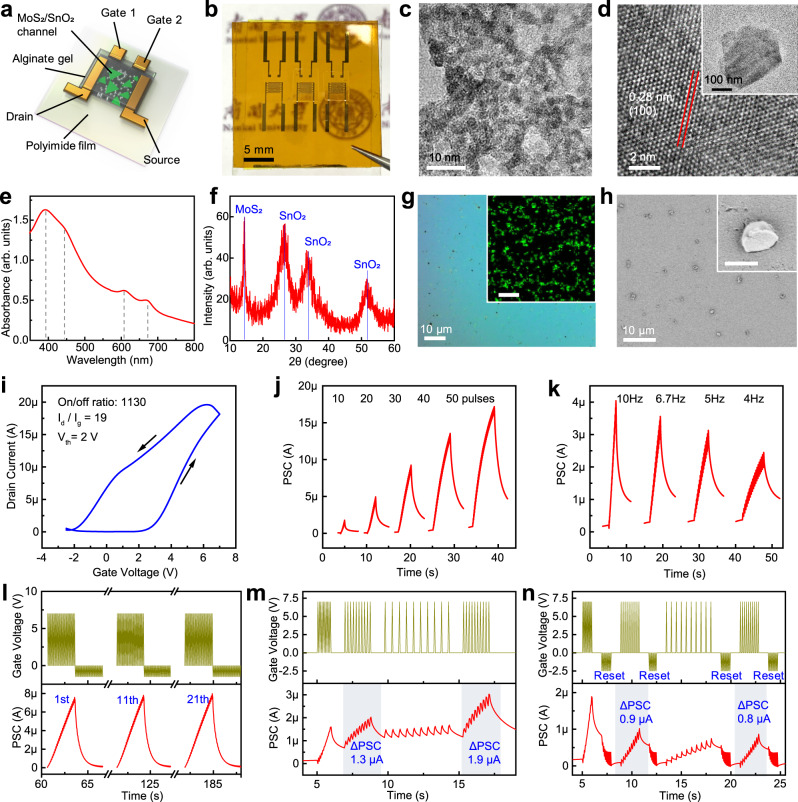
Characterization and performance of the synaptic transistor. **a** Schematic illustration of the device structure. **b** Photograph of the devices fabricated as an array on a flexible substrate. **c** TEM image of SnO_2_ nanoparticles. **d** High-resolution TEM of MoS_2_ nanoflakes showing that the measured lattice spacing can be indexed to (100) plane. Inset shows the TEM image of an individual MoS_2_ nanoflake. **e** UV–visible absorption spectra of MoS_2_ nanoflakes showing four absorption peaks, which are characteristics of transition metal dichalcogenides that have trigonal prismatic structure (2H phase). **f** XRD patterns of SnO_2_-nanoparticle/MoS_2_-nanoflake film. **g** Optical microscopy of the film. Inset shows Raman mapping acquired at a MoS_2_ peak of 408 cm^−1^ (scale bar: 10 μm). **h** SEM image of the film. Inset shows a close-up SEM image (scale bar: 500 nm). **i** Transfer curve of the device. *I*_d_, *I*_g_, and *V*_th_ represent drain current, gate leakage current, and threshold voltage, respectively. **j** Spike-number dependent plasticity of the device. PSC represents postsynaptic current. **k** spike-rate dependent plasticity of the device. **l** Cycle-to-cycle variation of the device. **m** BCM rule implemented by applying positive spike trains (7 V) with various frequencies to the device. ΔPSC represents a change in postsynaptic current. **n** Inhibition of the BCM rule by applying a negative spike train (−2.5 V) after each of the four positive spike trains to reset the device. Measurements in **i**–**n** were performed by applying voltage spikes to a single-gate (Gate1) of the device under a bias of 0.75 V.

The synaptic transistor (Fig. 2a) has a dual-gate structure composed of two lateral gates, alginate ion gel, nanoparticle-doped 2D-nanoflake channel (N-type), and interdigitated source and drain electrodes. The two lateral gates were coupled to the channel through the alginate ion gel^29,30^, and the final device (Fig. 2b) was fabricated by spin coating of SnO_2_-nanoparticle/MoS_2_-nanoflake on a flexible substrate. The ion gel is a soft hydrogel prepared from the natural material of sodium alginate through ionic crosslinking. The frequency-dependent capacitance of the ion gel (Fig. S2) exhibits a large capacitance of 1.03 μF cm^−2^ even at 1 kHz, which is attributed to the formation of electrical double layers (EDL) induced by mobile ions^31,32^. Formation of the EDL interfacial layer under applied voltage at high frequency is favorable for electrostatic modulation. The solution-processed fabrication protocol allows parallel and large-scale preparation of 100 device array on a 4-inch flexible substrate (Fig. S3).

The morphology of SnO_2_ nanoparticles (Fig. 2c) and MoS_2_ nanoflakes (Fig. 2d and Fig. S4) were studied using transmission electron microscopy (TEM). The greenish color of the MoS_2_ dispersion (Fig. S5) and the visible-range absorption of its UV–visible spectrum (Fig. 2e) indicate the presence of thin semiconducting MoS_2_ (2H phase)^33,34^. The photoluminescence spectrum of the MoS_2_ nanoflakes (Fig. S6) has a prominent peak at a photon energy of 1.9 eV, which is consistent with the band gap of layered MoS_2_. The thickness of the MoS_2_ nanoflakes is measured to be 6 nm, corresponding to nine layers (Fig. S7). To characterize the transistor channel, the mixture of SnO_2_ nanoparticles and MoS_2_ nanoflakes was spin-coated as a thin film on silicon. X-ray diffraction patterns (Fig. 2f) signify the presence of SnO_2_ and MoS_2_. Optical microscopy (Fig. 2g), Raman spectroscopy mapping at 408 cm^−1^ (corresponding to the A_1g_ peak of MoS_2_; Fig. 2g inset), and atomic force microscopy (AFM; Fig. S8) reveal that aggregates (up to 600 nm) of MoS_2_ nanoflakes are distributed evenly on the substrate surface. Scanning electron microscopy (SEM; Fig. 2h) indicates that the nanoflakes are formed as dispersed aggregates while the nanoparticles are closely packed as a continuous film with nanometer textures (Fig. S9).

The transistor characteristics of the device were first investigated. The transfer curve (Fig. 2i) shows large anticlockwise hysteresis (window of 4.5 V at 1 μA), which is caused by time lags in the migration and relaxation kinetics of mobile ions in the alginate gel^35^. The device has a threshold voltage (*V*_th_) of 2 V, which means that transistor–transistor logic (TTL) voltage of 5 V is sufficient to turn on the device. This device also shows a high on/off ratio (1130), low leakage current (*I*_d_/*I*_g_ = 19), and decent carrier mobility (*μ* = 0.3 cm^2^ V^−1^ s^−1^), and these characteristics (Fig. S10a) can compete with those of other electrolyte-gated flexible transistors^23,36^. In contrast, the transfer curve of the control device fabricated using pure SnO_2_ nanoparticles shows inferior device characteristics (Fig. S10b), including reduced mobility (*μ* = 0.06 cm^2^ V^−1^ s^−1^) and narrower hysteresis window (2.8 V at 1 μA). Such difference indicates that the MoS_2_ nanoflakes enhance carrier trapping under ion/electron electrostatic coupling, and yield improved characteristics, including mobility and electrostatic gating^37^. The influence of nanoflake size on the device performance (Fig. S11 and Table S1) is discussed in Supplementary Note 1.

The synaptic characteristics of the device were then studied. Drain current, defined as the postsynaptic current (PSC), is modulated by applying voltage spikes to one gate (Gate1). Positive spikes (7 V, 50 ms) have an excitatory effect, as demonstrated by the paired-pulse facilitation (PPF) effect (Fig. S12). Increasing the number or the frequency of positive voltage spikes leads to an increase in postsynaptic current, and thus spike-number dependent plasticity (SNDP; Fig. 2j) and spike-rate dependent plasticity (SRDP; Fig. 2k) are confirmed. Note that the control device fabricated using pure SnO_2_ nanoparticles shows deteriorated synaptic characteristics (Fig. S13). After stimulation by voltage spikes is stopped, the decay process of the postsynaptic current can be well-fitted to a memory loss curve (Fig. S14) with a typical time constant of 0.4 s, which is within the range of human sensory memory (0.2–3 s). Cycle-to-cycle variation (Fig. 2l) was evaluated by repeatedly applying 30 positive spikes and then 30 negative spikes, and the device exhibits multi-level conductance states (30), small cycle-to-cycle variation (5.3%), large conductance change (G_max_/G_min_ ~130), and linear potentiation (evaluated in Fig. S15), which are desirable properties for ideal synaptic devices^38^. The fabricated device shows small variations of synaptic response after being applied with 100 cycles of spike trains or stored in ambient conditions for 3 days (Fig. S16), indicating its high operation reliability and stability for long-term outdoor usage. Regarding the flexible characteristics, the synaptic properties of the device (Fig. S17) demonstrate high bending stability (<5% variation), thus enabling its potential applications in flexible electronics.

In neuroscience, the Bienenstock–Cooper–Munro (BCM) rule suggests that modulation of synaptic weight (the strength of a synapse) is frequency-dependent with tunable threshold, because it is influenced by the history of neural activities^39–41^. Accordingly, we implemented the BCM rule (Fig. 2m) in the device by applying four sets of spike trains (ten successive positive spikes) at frequencies of 10, 5, 2, and 5 Hz, respectively. The second and fourth spike trains, despite having the same frequency, cause different synaptic responses (ΔPSC) due to the influence of the previous (first and third) spike trains, so the synaptic plasticity of the device under such operation mode is history-dependent. In contrast, an additional application of negative spike trains (showing an inhibitory effect) after each of the four positive spike trains rapidly reduces the postsynaptic current to the resting level (Fig. 2n), and thus suppresses the BCM effect. Hence, synaptic plasticity can be tuned by such reset operation, and this is useful for adjusting the responsiveness of the device in an event-based manner. This device also shows potentiation behavior under stimulation by optical pulses, due to the UV-responsive properties of the SnO_2_ nanoparticles (Fig. S18), and the synaptic response of the device can be modulated by the intensity or the number of optical pulses (Fig. S19).

Overall, the usage of the 0D-nanoparticle/2D-nanoflake mixture enables solution-processable, low-temperature, scalable fabrication of flexible synaptic devices. Furthermore, the presented synaptic transistor is superior to other synaptic devices that use solution-processable 2D materials in terms of device characteristics and synaptic functions (Table S2).

**Fig. 3:**
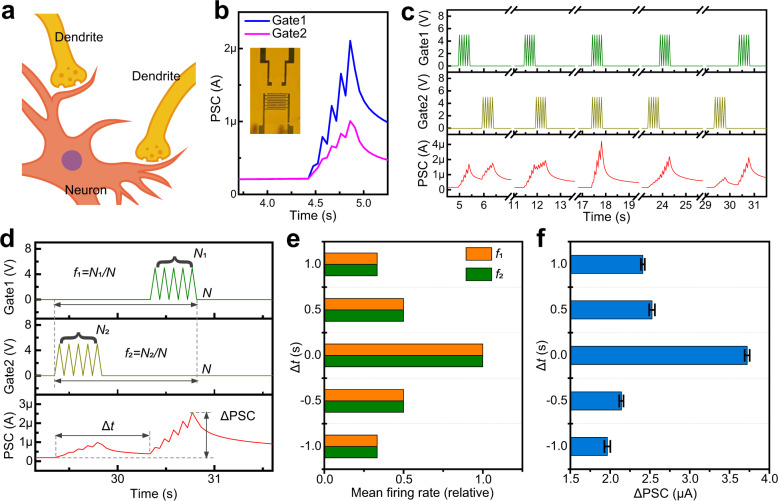
Spatiotemporal recognition capabilities of the synaptic transistor. **a** Illustration of a biological neuron with multiple dendrites. **b** Postsynaptic current measured by applying a spike train (5 V, 50 ms, 5 spikes) to the two gates respectively (Gate1: main gate; Gate2: minor gate). Inset shows a close-up photograph of the device. These two gates were assigned to different sensory modalities (assignment of perceptual weights). **c** Postsynaptic current (PSC) of the device when a series of pairwise spike trains with distinct timing (temporal delay Δ*t*: −1.0, −0.5, 0, 0.5, 1.0 s) were applied to the two gates (Gate1 and Gate2). **d** Definition of event duration (*N*; unitless), spike number (*N*_1_, *N*_2_), mean firing rate (*f*_1_, *f*_2_; relative value, unitless), and synaptic response (ΔPSC) under the stimuli of two spike trains with temporal delay (Δ*t*). Event duration was measured by spikes. **e** Mean firing rates of the two spike trains corresponding to the five cases in (**c**). **f** Synaptic response (ΔPSC) of the device corresponding to the five cases in (**c**). Spatiotemporal patterns of the two spike trains were well classified in multiple trials (*n* = 5), and the error bars in the figure represent the standard deviation (SD).

We then evaluated the device output in response to spatiotemporal spike trains. Our device emulates a multi-dendritic sensory neuron (Fig. 3a) that is capable of spatiotemporal integration, because its two lateral gates can receive sensory stimuli individually (Fig. 3b and Fig. S20). The gate close to the drain evokes a postsynaptic current that is approximately twice the current caused by the other gate close to the source, and the former and the latter gates, defined as a major gate (Gate1) and minor gate (Gate2), respectively, will be assigned to different sensory modalities depending on the specific applications. This is similar to the assignment of perceptual weights based on the reliability of sensory cues, which is observed in macaques during self-motion perception^25^. In our device, the perceptual weight can be modulated by exploiting the properties of spike-dependent plasticity or history-dependent plasticity (Fig. S21 and Table S3). Spatiotemporal recognition was implemented by applying two pairwise spike trains with distinct timing (temporal delay Δ*t*) to the two gates, and consequently, the postsynaptic current of the device (Fig. 3c) responded differently for these cases.

In a mammalian brain, head-direction cells are intricately linked to spatial memory and cognition, and they are classified into various types, such as regular spiking and fast-spiking, depending on their mean firing rates^42^. This time-averaging technique in neuroscience is adopted here for analyzing the characteristics of spike trains (Fig. 3d). An event that typically lasts several seconds dictates the duration of the spike stimulus to either gate of the device (Gate1 and Gate2), and the event duration is measured in units of spike duration, and then the mean firing rates (*f*_1_, *f*_2_; relative values) are derived by averaging the spike number over the event duration. Also, a synaptic response is quantified by the change in postsynaptic current (ΔPSC) after the occurrence of an event. The mean firing rates (Fig. 3e) and the synaptic response (Fig. 3f) were obtained for the five cases in Fig. 3c. The mean firing rate (*f*_1_ and *f*_2_) is regarded as a presynaptic characteristic, and it briefly represents the temporal delay between the two spike trains; however, it cannot identify the spike train that arrives first. The synaptic response (ΔPSC), regarded as a postsynaptic characteristic, can discern both the temporal difference and sequential order of the two spike trains, and thus it can accurately classify all the five spatiotemporal patterns, even considering its deviations obtained from multiple trials (error bars in Fig. 3f). Notably, simultaneous application of two spike trains with no temporal delay results in significantly high mean firing rates and synaptic response, revealing the facilitation effect of temporal-congruent spike trains. Above all, our device can parallelly process two input spikes with different spatiotemporal patterns, indicating its potential use for multisensory integration and spatiotemporal recognition. Besides, our dual-gate synaptic device differs from the previously reported multi-gate synaptic devices (which emulate hetero-synaptic and homeostatic plasticity under multiple gating modes) in terms of neural functions, fabrication protocol, mechanical flexibility, and applications enabled^43–45^.

### Motion-cognition of human activities

**Fig. 4:**
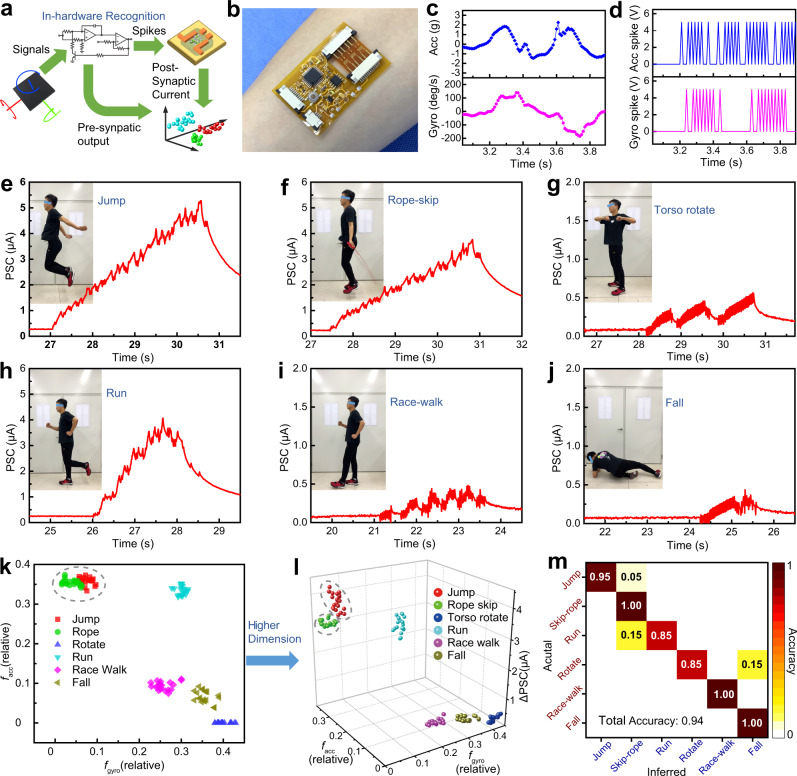
Application in human activity recognition. **a** Hardware diagram of the bioinspired neuromorphic motion-cognition nerve. Information flow during the execution of the motion recognition task is illustrated. **b** Photograph of the flexible synaptic electronics attached to human skin. **c** Acceleration (Acc) and gyroscopic (Gyro) signals acquired during an activity of running. **d** Encoded spike trains (Acc Spike, Gyro Spike) corresponding to the bimodal motion signals (Acc, Gyro) in (**c**). **e**–**j** Postsynaptic current (PSC) of the synaptic transistor when a human subject performed **e** jumping, **f** rope skipping, **g** torso rotation, **h** running, **i** race walking, and **j** falling. **k** Visualization of two hardware outputs (mean firing rate of acceleration spikes *f*_acc_, mean firing rate of gyroscopic spikes *f*_gyro_) in 2D coordinates. **l** Visualization of three hardware outputs (*f*_acc_, *f*_gyro_, synaptic response ΔPSC) in 3D coordinates, illustrating higher-dimensional classification strategy. The data clusters in six colors correspond to different activity types. **m** Confusion matrix showing the results of human activity recognition.

A bioinspired neuromorphic motion-cognition nerve (Fig. 4a) composed of an IMU sensor, spike-encoding circuit, and synaptic transistor was constructed. Both the spike-encoding circuit and the synaptic transistor were fabricated on flexible substrates, which can be attached to human skin (Fig. 4b). The IMU sensor and the spike-encoding circuit were both interfaced with a Bluetooth module to allow wireless transfer of motion signals, and this design could be used in a distributed sensor network. Details on multisensory integration are explained in the Methods. As for the implementation of neuromorphic electronics, our method using flexible synaptic transistors is beneficial over the conventional method using complementary metal–oxide–semiconductor (CMOS) devices in terms of circuit complexity, energy consumption, and device flexibility (Table S4), considering the pulse-drive, parallel operation of the former.

To prove the motion-cognition capabilities of our system, we performed experiments on human activity recognition^43^. The IMU sensor was attached to the chest of a human subject, who consecutively performed different activities with a typical resting time >2 s. For perceptual weighting, acceleration, and angular speed signals along vertical (gravity) direction were assigned to the major and minor sensory modalities, respectively. When no motion signals were detected after a waiting time (*T*_wait_) of 1.0 s, negative spikes were applied to reset the device (Supplementary Note 2). The bimodal motion signals acquired during an activity (e.g., running; Fig. 4c) were effectively converted to spike trains encoded with instantaneous frequency (Fig. 4d), and their temporal patterns, evaluated by correlation coefficient (Table S5)^46^, were well-preserved. In total, six types of human activities, including jumping, rope skipping, torso rotation, running, race walking, and falling were performed. Typical bimodal motion signals of the six activities are presented in Fig. S22a–f, and the corresponding postsynaptic currents are shown in Fig. 4e–j. The duration and amplitude of the postsynaptic current represent the event duration and the stimulus intensity, respectively. Repeatedly performing each of the six activities completed a training process (Fig. S23), and the hardware outputs were recorded to derive the decision boundaries for classifying activity types. When presynaptic signals, including mean firing rates of acceleration and gyroscopic spikes (*f*_acc_ and *f*_gyro_) were used for classification (Fig. 4k), some activity types overlapped (e.g., jumping and rope skipping) and cannot be classified. In contrast, when presynaptic and postsynaptic signals (*f*_acc_, *f*_gyro_, and ΔPSC) were utilized and mapped to higher-dimensional space (Fig. 4l), all the six activity types were distinct from each other (decision boundaries: Table S6). Therefore, to ensure accurate classification, the three hardware outputs (*f*_acc_, *f*_gyro_, and ΔPSC) were used to derive the decision boundaries. Following the training process, a testing process (Figure S24) was implemented by performing different activities, and the corresponding activity types were recognized by comparison operations using a decision-tree method (Fig. S25). The experimental results presented as a confusion matrix (Fig. 4m) shows that the total recognition accuracy is as high as 0.94 (classification results with and without using ΔPSC are compared in Fig. S26 and Table S7). Such performance of our system can rival those of previously reported human-motion perception systems (Table S8), which instead use processing units. The accuracy of motion recognition can be further improved (Supplementary Note 3) by optimizing the decision boundaries (Fig. S27) or changing the sensor type (Figs. S28–S31). In addition, the human motion recognition task has been performed using multiple devices fabricated in the same batch, and the similar synaptic response of different devices (Figs. S32,  S33) as well as their close value of recognition accuracy (Table S9) suggest that the motion-cognition method is robust.

We further performed experiments on the classification of flag semaphore signals, which involve complicated motion sequences (Fig. S34). Considering that flag-waving motion generates temporally correlated signals of rotational inertia and angular speed (Fig. S35), the profile of the postsynaptic current (Fig. S36) depicts the duration, sequence, and intensity of such motion. Flag semaphore was tested for all 26 letters, and the corresponding hardware outputs (*f*_acc_, *f*_gyro_, and ΔPSC; typical values) visualized in 3D coordinates (Fig. S37) show that the data points representing the 26 letters can be distinguished from each other (decision boundaries: Table S10). In addition, our system also successfully achieves the classification of hand gestures (Figs. S38, S39 and Table S11), indicating its potential application in the smart interface.

### Motion-cognition for aerial robots

**Fig. 5:**
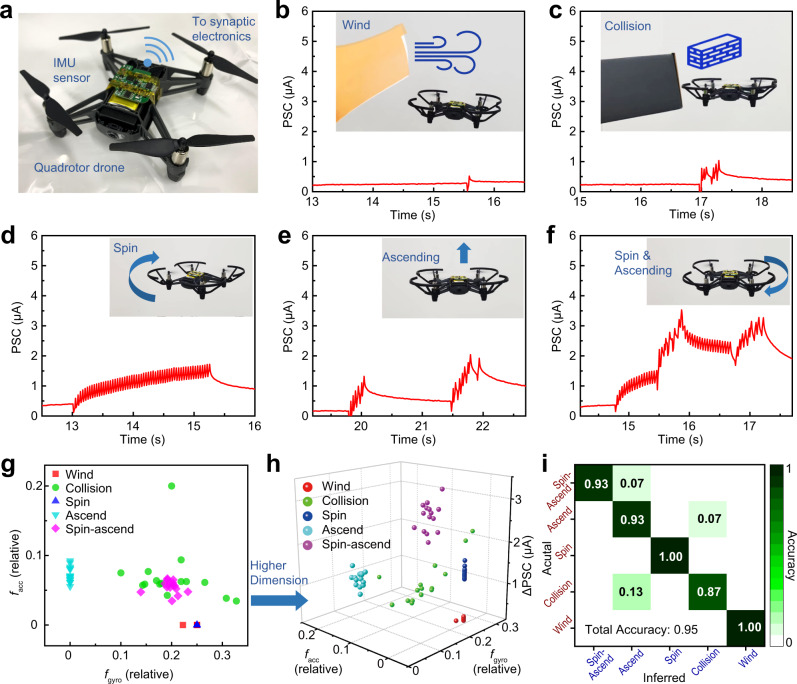
Application in recognition of drone flight modes. **a** Motion-cognition quadrotor drone equipped with the IMU sensor, which wirelessly transmits bimodal motion signals to the spike-encoding circuit and synaptic transistor. Recognition of drone flight modes was implemented by spike coding and synaptic processing of the motion signals. **b**–**f** Postsynaptic current (PSC) generated from the synaptic transistor, when the drone was operated in five modes: **b** wind stabilization, **c** collision stabilization, **d** spinning, **e** ascending, and **f** spin-ascending. **g** Visualization of the two hardware outputs (mean firing rate of acceleration spikes *f*_acc_, mean firing rate of gyroscopic spikes *f*_gyro_) in 2D coordinates. **h** Visualization of the three hardware outputs (*f*_acc_, *f*_gyro_, and synaptic response ΔPSC) in 3D coordinates. The data clusters in different colors correspond to the five flight modes. **i** Confusion matrix showing the results of drone flight-mode recognition.

To demonstrate cognitive perception capabilities towards robotic intelligence, our system was tested using a quadrotor drone (Fig. 5a). For perceptual weighting, an acceleration signal along vertical (gravity) direction was assigned to the major sensory modality. Recognition of drone flight modes was implemented through training and testing processes (Fig. S40) using hardware outputs, similar to before. A preliminary experiment was performed by executing a spin-ascending operation, which involves constant rotation coupled with sudden acceleration and deceleration. The detected motion signals (Fig. S41a) and the encoded spike trains (Fig. S41b) both show that acceleration and angular speed were correlated during this operation. Further experiments were conducted by operating the drone in different flight modes, including wind stabilization, collision stabilization, spinning, ascending, and spin-ascending. Motion signals (Fig. S42a–e) and postsynaptic current (Fig. 5b–f) acquired under these five flight modes were recorded. Repeated execution of each of the five flight modes completed the training process (waiting time *T*_wait_ 1.5 s). Again, visualizations of the hardware outputs in 2D (*f*_acc_, *f*_gyro_; Fig. 5g) and 3D coordinates (*f*_acc_, *f*_gyro_, and ΔPSC; Fig. 5h) reveal that higher-dimensional classification makes it easier to differentiate drone flight modes (decision boundaries: Table S12). The confusion matrix (Fig. 5i) indicates that the total accuracy of this recognition task reaches 0.95 (classification results with and without using ΔPSC are compared in Fig. S26 and Table S7). The performance of our synaptic-electronics-enabled system in the detection/recognition of drone motion can rival those of the sensor-processor systems, which instead rely on complicated algorithms and computational resources (Table S13). Moreover, the drone motion classification task has been executed using multiple devices (Figs. S32, S33), and the experimental results show that the recognition accuracy is quite high with small variations among different devices (Table S9). Overall, the high accuracy and robustness of the motion-cognition mechanism can be attributed to the low device-to-device variation benefitted from the reliable fabrication route, the effectiveness of the multisensory integration approach, and the combined usage of presynaptic and postsynaptic signals for classification.

### Multisensory integration and perceptual enhancement

By integration of optical flow and vestibular acceleration cues, macaques show improved behavioral response in motion and spatial perception tasks under bimodal sensory stimuli (sensory inputs from two modalities are integrated) compared to that under unimodal sensory stimuli (each sensory input is segregated)^6,25^. Therefore, to quantify the magnitude of cross-modal perceptual enhancement in our system, we adopted a multisensory enhancement index (MEI), which is used in multisensory perception in neuroscience:

1 $${{{{{\rm{MEI}}}}}}=\frac{{R}_{{{{{{\rm{bimodal}}}}}}}-{{\max }}({R}_{{{{{{\rm{modal}}}}}}1},\,{R}_{{{{{{\rm{modal}}}}}}2})}{{{\max }}({R}_{{{{{{\rm{modal}}}}}}1},\,{R}_{{{{{{\rm{modal}}}}}}2})}$$

where *R*_bimodal_ signifies the recognition accuracy under the bimodal sensory stimuli condition, and *R*_modal1_ and *R*_modal2_ represent the recognition accuracy under unimodal sensory stimuli conditions^47^. Additional control experiments were performed by applying unimodal sensory stimuli (acceleration or angular speed) to the synaptic transistor, and executing motion recognition tasks under unimodal sensory conditions (Figs. S43, S44). The results (Table S14) reveal that the recognition accuracy is higher under bimodal sensory stimuli than under unimodal sensory stimuli, and MEI enhancement due to cross-modal integration reaches 15% for human activity recognition and 25% for drone flight-mode recognition. Thus, cross-modal perceptual enhancement regarding motion-cognition was confirmed in our system.

Another essential principle of multisensory integration in neuroscience is inverse effectiveness, which describes that cross-modal perceptual enhancement is large under weak stimuli and decreases as stimulus intensity increases^26^. We further examined the bimodal perceptual enhancement of our system in terms of different human activity types (Fig. S43). The largest increase in recognition performance was observed in the case of falling motion, corresponding to the weakest stimulus due to its shortest event duration and lowest synaptic response. A similar rule was also observed in the recognition of drone flight modes (Fig. S44). Thus, an inverse relationship between multisensory integration and unisensory responsiveness was identified^4^.

As an extension, multisensory integration of other sensory cues, which were obtained from completely different sensors, was investigated by using our system. Implementing the sensing modules using an optical-flow sensor, vibrotactile sensor, and IMU sensor allows the detection of multimodal signals corresponding to visual, tactile, and vestibular cues (Fig. S28). Bimodal sensory integration of vestibular and visual cues results in a recognition accuracy of 0.96 for human activities (Fig. S29). Similarly, bimodal sensory integration of vestibular and vibrotactile cues leads to a recognition accuracy of 0.95 for human activities (Fig. S30). Compared with unimodal sensory conditions where each sensory cue is segregated (Fig. S31), bimodal sensory integration of different cues improves recognition accuracy and achieves perceptual enhancement (Tables S15, S16 and Supplementary Note 4). By connecting to desired sensing modules (vestibular, optical, and vibrotactile), our system can be potentially utilized as a general neuromorphic platform for the emulation of multisensory neural processing in the brain, ranging from cue integration to causal inference.

## Discussion

In summary, we demonstrate a flexible multisensory synaptic transistor and further develop a bioinspired neuromorphic motion-cognition nerve, which emulates multisensory integration of ocular-vestibular cues in macaques. This system simultaneously achieves neuromorphic intelligence, cognitive function, and perceptual enhancement toward motion perception. A fast, scalable, and solution-processed approach is developed for the fabrication of nanoparticle-doped 2D-nanoflake thin film. Benefited from the charge-trapping effect of the 2D material, a high-performance flexible neuromorphic device was then fabricated by using this thin film as a semiconductor channel. This device exhibits improved electrical properties of carrier mobility and electrostatic gating, and superior synaptic characteristics of history-dependent plasticity and spatiotemporal recognition capability, which enable parallel, synaptic, event-based processing of multisensory inputs. Using the presynaptic and postsynaptic signals as classification criteria, recognition of human activity types and drone flight modes were successfully demonstrated with total accuracy of 0.94 and 0.95, respectively. Furthermore, bimodal sensory integration of visual, tactile, and vestibular cues implemented by changing the sensing module in our system all lead to the reliable and high-accuracy performance of motion recognition. Essentially, this system is biologically plausible, because it emulates cross-modal integration of sensory cues, and realizes cognitive functions using presynaptic and postsynaptic signals, and its perceptual performance matches the biological principles of perceptual enhancement by multisensory integration. Therefore, our system provides a new paradigm for combining cognitive neuromorphic intelligence with multisensory perception towards applications in sensory robotics, smart wearables, and human-interactive devices.

## Methods

### Materials

Few-layer MoS_2_ nanoflakes (2H phase, 1 mg ml^−1^) prepared by liquid-phase exfoliation and dispersed in water were customized and purchased from XFNano. Ethanol was added into the dispersion (v/v 1:4) to reduce the surface tension. The nanoflake dispersion was sonicated for 30 min in an ultrasonic bath, then centrifuged for 5 min at 500 × *g*, and the decanted supernatant was sonicated again for 15 min. The lateral size of the MoS_2_ nanoflakes was measured to be 200–600 nm. Colloidal dispersion (15%) of SnO_2_ nanoparticles in water was purchased from Alfa Aesar, and the average particle size was measured to be ~4 nm. Sodium alginate, acetic acid, and glycerol were purchased from TCI.

### Device fabrication

To fabricate the semiconducting film, a mixture (v/v 10:1) of MoS_2_ nanoflake dispersion and SnO_2_ nanoparticle dispersion was sonicated for 15 min, then spin-coated at 1000 rpm on a clean polyimide substrate, which was then baked at 80 °C for 1 min. The spin-coating process was repeated twice to ensure full coverage of the sample surface, then the sample was annealed at 110 °C for 30 min in a nitrogen-filled glove box. Source, drain, and dual planar gates were subsequently deposited by thermal evaporation using a shadow mask that defines an interdigitated channel with a length of 80 μm. Alginate solution was obtained by adding sodium alginate powder (0.06 g) to an acetic acid solution (2 wt%, 4 ml), then stirring the mixture at 600 rpm at 70 °C for 10 min. As the alginate dissolved, glycerol (0.2 g) was added dropwise. After cooling to room temperature, the viscous alginate solution was drop-cast on a clean glass slide, then allowed to dry at 80 °C for 40 min, and this process yielded an ion gel. The final device was obtained by covering the regions of channels and gates using the ion gel that was cut to the desired size. The device can operate in single-gate or dual-gate mode, and its synaptic response can be modulated by electrical or optical pulses.

### Multisensory integration

A commercially available IMU module (BWT901, WitMotion) was used as the sensor, and it includes an accelerometer (XYZ axis), a gyroscope (XYZ axis), a magnetometer, and a Bluetooth module (for wireless communication). Bimodal motion signals of acceleration and angular speed were simultaneously acquired from the sensor at a sampling rate of 100 Hz, and the signals were further transmitted to a spike-encoding circuit through Bluetooth. The spike-encoding circuit was implemented in a custom-built flexible printed circuit board (FPCB) integrated with a low-power microcontroller (ATmega328P, Microchip), which was programmed to convert the bimodal motion signals into two spike trains (5 V) with spatiotemporal patterns (pseudocode for spike conversion is shown in Supplementary Note 5). Acceleration and angular speed signals at one axis were encoded, and spike-rate coding with thresholding was adopted to set the instantaneous frequency (25 or 50 Hz) of the spike trains. This coding strategy ensures that the temporal patterns of the input signals are well preserved after spike conversion. Subsequently, the two spike trains corresponding to two sensory modalities (i.e., inertia and speed) were sent to the two gates of the synaptic transistor. Finally, the synaptic transistor generated a postsynaptic current under the concurrent stimuli of two spike trains. This process achieves the parallel and efficient integration of two sensory cues in our neuromorphic motion-cognition system, which emulates the multisensory integration process in the mammalian brain. Moreover, different sensory cues can be obtained by replacing the sensor in our system. For example, building the sensing module using optical-flow, vibrotactile, and accelerometer sensors achieves the acquisition of ocular, tactile, and vestibular sensory cues, respectively. Bimodal sensory integration of two different sensory cues (including integration of visual-vestibular cues and integration of vibrotactile-vestibular cues) have been realized using our system (Supplementary Note 4).

### Motion-cognition setup

Recognition of human activities, including body movement, flag semaphore signaling, and hand gestures, were performed by adhering the IMU sensor to the skin (chest, forearm, hand) of an adult subject. Recognition of drone flight modes was conducted by mounting the IMU sensor on a slightly modified quadcopter drone (Tello, Ryze Tech; weight 84 g). The bimodal motion signals acquired by the IMU sensor were wirelessly sent to the spike-encoding circuit through Bluetooth, encoded as two voltage spike trains, and processed by the synaptic transistor. The device was reset by negative spikes when no motion signals were detected after a waiting time (*T*_wait_). The reset operation of the device was achieved by using a capacitor-diode circuit (Fig. S45), which generates negative voltage spikes (Supplementary Note 2), while the signal-detection function was directly implemented in the microcontroller, which can be further replaced by a simple circuit. In this way, the device could operate in a continuous, event-based manner. To fulfill the recognition tasks, a training process was executed to obtain decision boundaries for classification. Three hardware outputs, including mean firing rates of the acceleration and gyroscopic spikes (*f*_acc_ and *f*_gyro_) and the synaptic response of the device (change in postsynaptic current ΔPSC), were recorded in terms of the corresponding motion type. The mean firing rate (relative value) was defined as the ratio of spike number to event duration, and event duration (unitless) was counted in units of the shortest spike duration. The decision boundary was simply set as the midpoint of two neighboring levels (explained in Supporting Information). Afterward, a testing process was performed to predict the motion type using the decision-tree method. The decision-tree method (explained in Supporting Information), which compares the hardware outputs (*f*_acc_, *f*_gyro_, and ΔPSC) with their decision boundaries, was implemented in the microcontroller for the recognition of motion signals. The signal flow of our system is schematically illustrated in Fig. S46, and details on data recording, data processing, and parameter detection are explained in Supplementary Note 6.

### Characterization and measurement

Characterizations of the nanomaterials were carried out using a scanning electron microscope (Apreo-S, Thermo Fisher), a transmission electron microscope (Talos F200X G2, Thermo Fisher), an atomic force microscope (Dimension Icon, Bruker), an X-ray diffractometer (Ultima IV, Rigaku), ultraviolet-visible spectroscopy (Cary 5000, Agilent), a fluorescence spectrometer (FS5, Edinburgh Instruments), and a confocal Raman imaging system (XperRAM 200, NanoBase) having optical microscopic imaging and Raman mapping functions. The properties of the ion gel were analyzed using a semiconductor device analyzer (B1500A, Agilent). Electrical characteristics of the synaptic transistor were measured using a probe station equipped with a semiconductor analyzer (4200-SCS, Keithley). The optical response of the synaptic transistor (without ion gel) was measured under optical spikes generated from a Xenon light source (Gloria, Zolix) at 250 nm wavelength.

Signed informed consent have been obtained from the human subjects to use the images and conduct the experiments described in this work. This study (protocol no. NKUIRB2023003) was approved by the Institutional Review Board at Nankai University.

## Supplementary information

Supplementary Information

## Data Availability

The data generated in this study are provided in the Supplementary Information and Source Data file, or from the corresponding author upon reasonable request. Source data are provided with this paper.

## Source data

Source Data

## Code availability

The code for the spike conversion function and decision-tree method are available from the corresponding author with detailed explanations upon reasonable request.
