# Supplementary material for: Mammalian-brain-inspired neuromorphic motion-cognition nerve achieves cross-modal perceptual enhancement
Source: Nat Commun. 2023 Mar 11;14:1344. doi: 10.1038/s41467-023-36935-w (PMC10008641; doi:10.1038/s41467-023-36935-w)
Supplement: Supplementary file 1 — Supplementary Information [file 41467_2023_36935_MOESM1_ESM.pdf]

Supporting Information

Mammalian-brain-inspired neuromorphic motion-cognition nerve achieves cross-modal perceptual enhancement

Chengpeng Jiang[1,2,3], *Jiaqi Liu*[1,2], *Yao Ni*[1,2], *Shangda Qu*[1,2], *Lu Liu*[1,2], *Yue Li*[1,2], *Lu Yang*[1,2], *Wentao Xu*[1,2]*

[1] Institute of Photoelectronic Thin Film Devices and Technology, Key Laboratory of Photoelectronic Thin Film Devices and Technology of Tianjin, College of Electronic Information and Optical Engineering, Engineering Research Center of Thin Film Photoelectronic Technology of Ministry of Education, School of Materials Science and Engineering, Smart Sensing Interdisciplinary Science Center, Nankai University, Tianjin 300350, China.

[2] Shenzhen Research Institute of Nankai University, Shenzhen 518000, China.

[3] Research Center for Intelligent Sensing, Zhejiang Lab, Hangzhou 311100, China.

*Corresponding author. E-mail: wentao@nankai.edu

Supplementary Note 1

Influence of nanoflake size on device performance

The size of the MoS_2 nanoflakes are changed while the size of the SnO_2 nanoparticles are unchanged in preparation process of the semiconducting materials, in order to investigate the influence of MoS_2 nanoflake size on the performance of the synaptic transistor. MoS_2 nanoflakes with different sizes were produced using a size selection procedure to separate large-size material and small-size material. Small-size MoS_2 nanoflakes (lateral size: 100–300 nm) and large-size MoS_2 nanoflakes (lateral size: 400–700 nm) were utilized to fabricate two different synaptic devices respectively. Performance of the first device fabricated using small-size nanoflakes and the second device prepared using large-size nanoflakes are presented in Figure S11. Their device parameters are compared in Table S1. Large-size nanoflake improves some device parameters, including hysteresis window, threshold voltage, and turn-on current, but it also compromises other device parameters, including leakage current and signal-to-noise ratio. Thus, all the device parameters related to transistor and synaptic characteristics should be well considered when tailoring the size of the MoS_2 nanoflakes.

Supplementary Note 2

Reset operation of the synaptic device

In principle, the reset operation of the synaptic device is achieved by using a simple circuit with charging and discharging functions, as illustrated in Figure S45. This circuit includes two diodes (D1, D2) and two capacitors (C1, C2). The input pin of this circuit is directly connect to the digital output pin of the microcontroller (MCU) in our system. The output pin of this circuit is connected to the gate of the synaptic device. At first, the presence of positive signal (+5V) at the input pin results in the formation of charging branch of C1 and D1. Subsequently, when the positive signal at the input pin is reset to zero (falling edge), discharging branch of C1, C2 and D2 is formed. In this way, this circuit achieves the generation of negative voltage spikes close to −2 V, and the peak value and width of the negative spikes can be adjusted by tuning the forward voltage drop of the two diodes or the capacitance value of the two capacitors. Noteworthy, this circuit is fully compatible with our neuromorphic system, considering that the microcontroller only generates positive signal of +5 V and the synaptic device requires reset spikes of around −2 V.

Supplementary Note 3

Further improvement of the motion-recognition accuracy

We propose two methods for further improving the recognition accuracy of human motion types.

In the first approach, the decision boundaries for motion types are optimized. Specifically, the decision boundary for distinguishing run and rotate motions is set based on the firing rate of gyroscopic signal (f_{gyro}) instead of the postsynaptic current change (ΔPSC), and the level of the decision-tree method is increased from one to two, corresponding to a two-level decision making procedure, as schematically shown in Figure S27a. The results of human motion classification as presented in Figure S27b reveal that the total accuracy for human motion recognition is 0.97. Thus, this approach of decision-boundary optimization achieves the improvement of recognition accuracy.

In the second approach, the type of the sensor for acquiring human motion signals is changed in order to obtain totally different sensory cues. As shown in Figure S28a, an optical sensing module has been built. The bimodal motion signals of acceleration and optical-flow speed are acquired by using the accelerometer and the optical sensing module, respectively. Then, the bimodal motion signals are encoded into two spike trains and processed by the synaptic transistor, achieving the multisensory integration of vestibular and ocular cues. The experimental results in Figure S29 reveal that the total accuracy for human motion recognition is 0.96. Hence, this approach of vestibular-ocular sensory integration also achieves the improvement of recognition accuracy.

Supplementary Note 4

Multisensory integration of different sensory cues

Multisensory integration of totally different sensory cues, such as visual, tactile, or vestibular, have been implemented in the neuromorphic motion-cognition system.

First of all, it should be noted that the synaptic device only shows potentiation response to optical stimuli that have ultraviolet wavelength (e.g., 250 nm) and short spike duration (e.g., 50 ms). In contrast, the light source (including the sunlight and indoor light) that induce ocular response have visible-range wavelength and are continuous in the time domain. Therefore, an additional optical sensing module has been built, as shown in Figure S28a, in order to detect the visible-wavelength, temporal-continuous optical stimuli for obtaining ocular cue. This optical sensing module is built from photo detector array and modulatory resistors. Moreover, to obtain tactile cue, a vibrotactile sensing module is customized by mounting PVDF piezoelectric film on a 3D-printed block, adding proof mass on top of the encapsulated PVDF film, and connecting to a charge amplifier circuit, as shown in Figure S28b. This module with cantilever structure is sensitive to external vibrations, and hence vibrotactile signal can be detected. As show in Figure S28c, the final implementation of the sensing unit in our system comprises the optical, the vibrotactile, and the vestibular sensing modules. The three sensing modules which obtain visual, tactile and vestibular sensory cues are mounted onto a flexible fabric, and can be attached to the chest of human subject.

Additional experiments on human activity recognition were conducted by using motion signals from different sensors, and then two sensory cues were encoded as two spike trains and concurrently processed by the synaptic device for the recognition of human activity type. The experimental results of human motion classification are presented as confusion matrix. As shown in Figure S29, bimodal sensory integration of vestibular and optical cues results in a high recognition accuracy of 0.96. Similarly, as presented in Figure S30, bimodal sensory integration of vestibular and vibrotactile cues leads to a high recognition accuracy of 0.95. Control experiments reveal that the recognition accuracy under single sensory input of vestibular, optical, and vibrotactile are 0.825, 0.708 and 0.858, as shown in Figure S31. Therefore, the cross-modal integration of two different sensory cues results in improvement of recognition accuracy regarding motion cognition. Furthermore, the multisensory enhancement effect has been examined by calculating the multisensory enhancement index (MEI). As shown in Table S15, the MEI value reaches 16.12% when vestibular and optical sensor cues are integrated. Likewise, Table S16 reveals that the MEI value reaches 10.68% when sensory integration of vestibular-vibrotactile cues are implemented.

These results demonstrate that our neuromorphic motion-cognition system achieves the multi-sensory integration of totally different sensory cues such as visual, tactile, and vestibular, which are obtained from different sensors. Moreover, the recognition accuracy of human motion under bimodal sensory condition where sensory cues are integrated is much higher than under unimodal sensory conditions where sensory cues are segregated, and those results indicate that perceptual performance is enhanced due to multisensory integration.

Supplementary Note 5

Pseudocode for the spike-conversion function

In our neuromorphic motion-cognition system, the spike-conversion function is realized by programming the microcontroller using simple software code. The pseudocode for this spike-conversion function is provided as follow:

```
Define threshold level 1 for input signal as SIGNAL_LEVEL1, for example 0.2 g
Define threshold level 2 for input signal as SIGNAL_LEVEL2, for example 0.4 g
Define spike duration time as DELAY0, for example 10 ms

Main Loop of spike conversion function ( )
{
    read sensor signal and store it as Input_Signal
    if (Input_Signal > SIGNAL_LEVEL2)
    {
        generate spike sequence of "1" "0" with duration time of DELAY0
    }
    else if (Input_Signal > SIGNAL_LEVEL1)
    {
        generate spike sequence of "1" "0" "0" "0" with duration time of DELAY0
    }
    else
    {
        generate spike of "0" with duration time of DELAY0
    }
}
```

In this code, different threshold levels for the input signal are defined at first. For example, regarding the vestibular acceleration signal measured from the inertial sensor, threshold level 1 and 2 are defined. Then, spike duration time is defined as well, considering the sampling rate of the sensor signal. The main loop of the spike-conversion function starts with reading the present value of sensor signal (i.e., *Input_Signal*), and proceeds to compare the sensor signal with the predefined threshold levels, and finally generates spike sequence by employing the spike-rate coding strategy. Briefly, higher magnitude of the sensor signal corresponds to higher instantaneous frequency of the encoded spikes. In this way, the sensor signal is effectively converted to spike trains encoded with instantaneous frequency, achieving the spike conversion-function.

Supplementary Note 6

Details on data recording, data processing, and parameter detection

In our current design of the neuromorphic motion-cognition system, the recorded data are the presynaptic spike trains from the spike-encoding circuit and the postsynaptic current from the synaptic device. The bimodal motion signals acquired from the sensor are wirelessly sent to the spike-encoding circuit, then encoded into two spike trains, and finally processed by the synaptic transistor. Presynaptic signals of the two spike trains are recorded using the microcontroller. Postsynaptic signal of the postsynaptic current is recorded using the semiconductor analyzer. Then, the three hardware outputs including mean firing rates of the acceleration/gyroscopic spikes and synaptic response of the device are derived for each motion type, and motion-cognition tasks are performed by comparing the hardware outputs with the decision boundaries of motion type. The parameters detected during measurements of motion signals are the physical properties regarding the translational, rotational, or vibrational movement of the object. In the original design using the IMU sensor, the parameters measured from the sensor are the acceleration (in unit of standard acceleration denoted as g) and angular speed (in unit of degree/s) of motion. In the extended design using IMU, optical, and tactile sensors, the parameters measured from the sensor include the acceleration, optical flow speed, and vibration of motion.

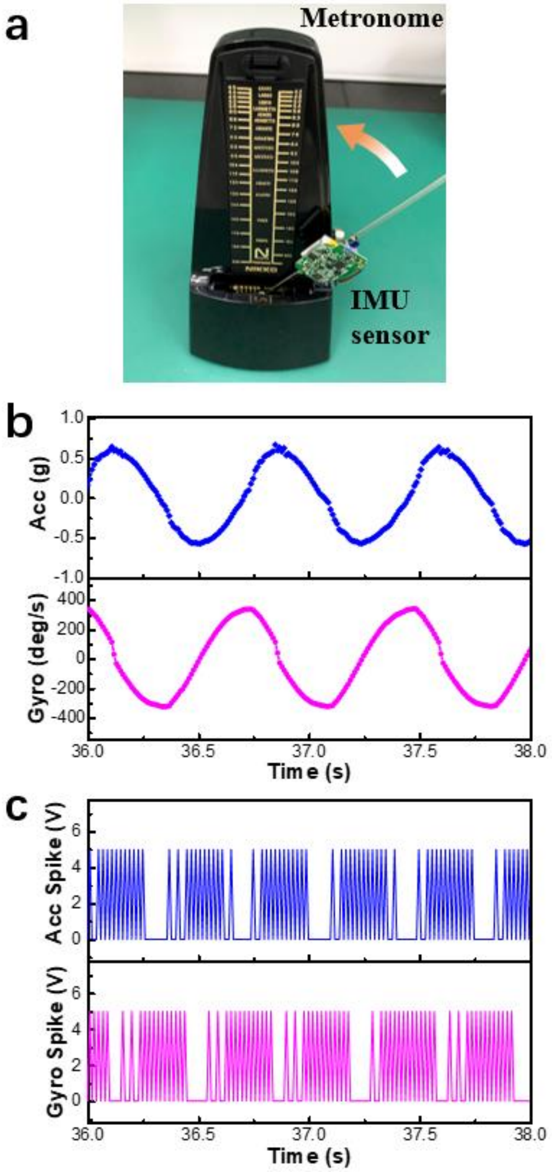

Figure S1. Motion sensing performance of the IMU sensor and spike encoding of the as-measured bimodal motion signals. (a) Motion sensing test conducted by mounting the IMU sensor on a mechanical metronome, which swung around a pivot at a fixed frequency. (b) Bimodal motion signals of acceleration (Acc) and gyroscopic angular speed (Gyro) measured from the IMU sensor. Both signals show sinusoidal profiles with constant amplitude and frequency, reflecting the harmonic pendulum-like motion of the metronome. (c) The encoded spike trains corresponding to the bimodal signals (Acc and Gyro) in (b). The bimodal motion signals are converted to two spike trains that have instantaneous frequency (i.e., instantaneous firing rate) proportional to the signal's amplitude. This spike-rate coding method ensures that the temporal patterns of the two signals are well preserved after converted into spikes, because their correlation coefficient (calculated in Table S2) is maintained.

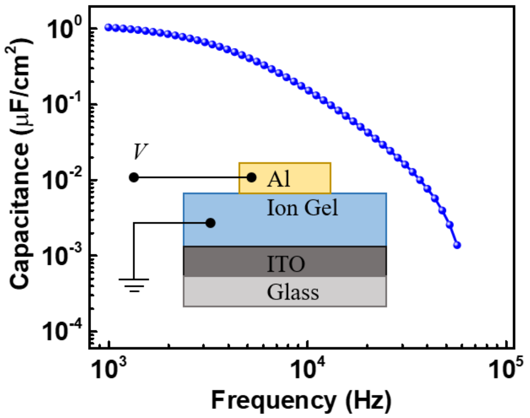

Figure S2. Frequency-dependent specific capacitance of ion-gel film of sodium alginate. The alginate gel was sandwiched between indium tin oxide (ITO) and Al electrode. Inset shows that the structure of the test sample is Al/ion-gel film/ITO glass.

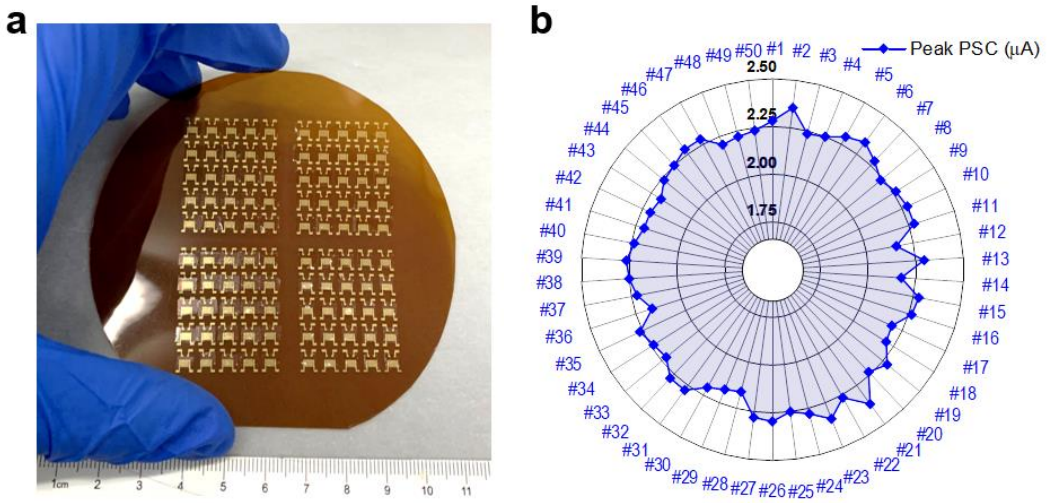

Figure S3. Wafer-scale fabrication of synaptic transistor array. (a) Device array of 100 synaptic transistors fabricated on a flexible polyimide substrate with 4-inch diameter. (b) Variations of peak postsynaptic current among 50 different devices.

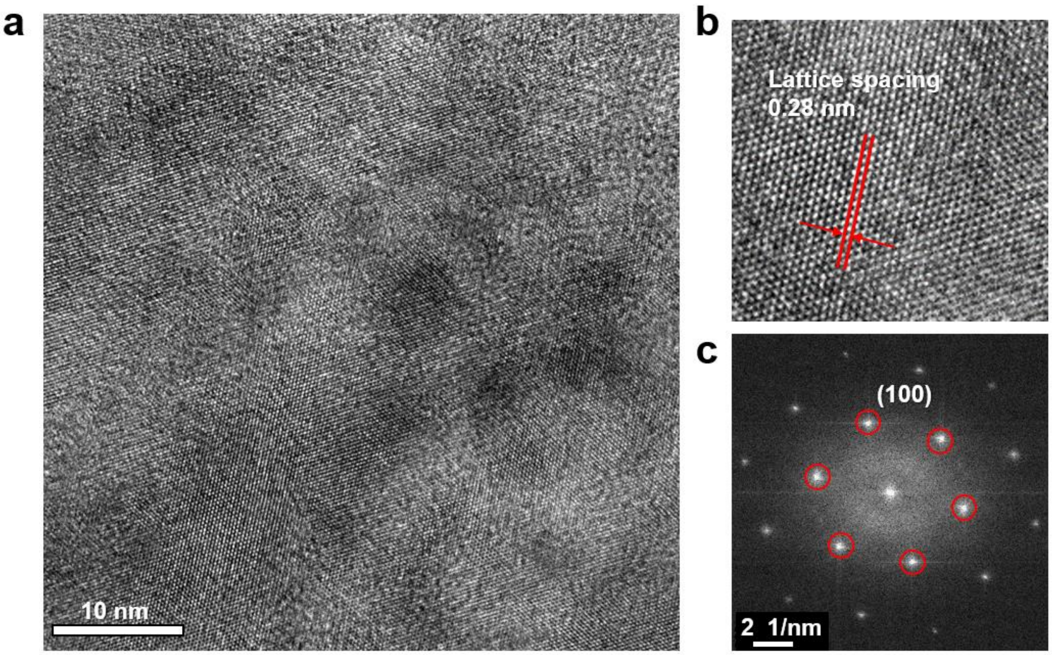

Figure S4. High-resolution transmission electron microscopy (HR-TEM) characterization of MoS_2 nanoflakes. (a) HR-TEM image showing the crystalline structure of MoS_2 nanoflakes. (b) Magnified HR-TEM image revealing the atomic arrangement of MoS_2. It is confirmed that the labelled plane with 0.28 nm spacing can be well indexed to the (100) plane of MoS_2. (c) Selected-area electron diffraction image corresponding to the HR-TEM image in (a). The six bright diffraction spots in radial symmetry, marked in red circles, indicate the highly crystalline nature of the MoS_2 nanoflakes.

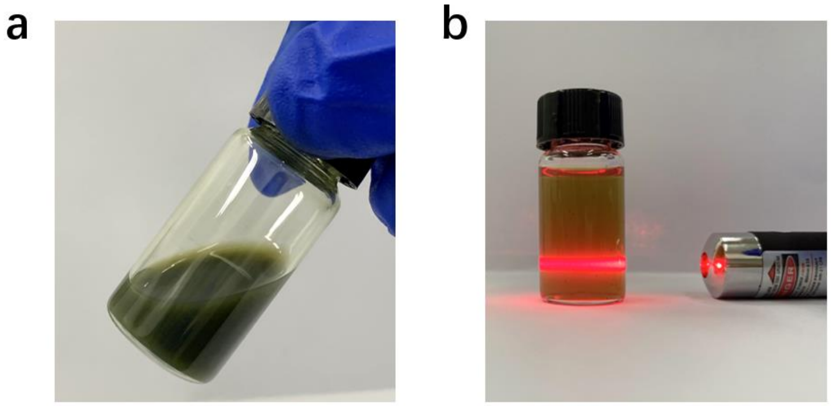

Figure S5. Photograph of MoS_2 nanoflake dispersion. (a) Photograph of MoS_2 nanoflakes dispersed in ethanol/H_2O, showing that the dispersion is in dark green with high concentration (1 mg/ml). The greenish color of the dispersion indicates the presence of thin and semiconducting MoS_2 (2H phase), because bulk (not layered) or metallic (1T phase) MoS_2 crystals have featureless absorption in the entire visible-light range and thus appear black. (b) Diluted MoS_2 dispersion (0.1 mg/ml) in greenish yellow color displaying the Tyndall effect, which indicates the formation of stable colloidal dispersion of MoS_2.

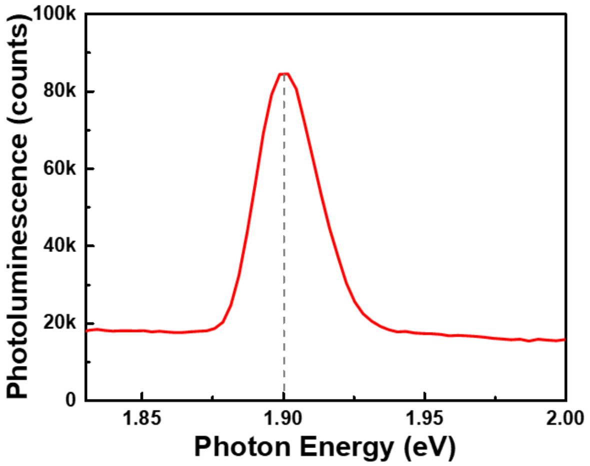

Figure S6. Photoluminescence spectra of MoS_2 nanoflakes. The significant peak at ~1.9 eV photon energy is consistent with the band gap of few-layer MoS_2.

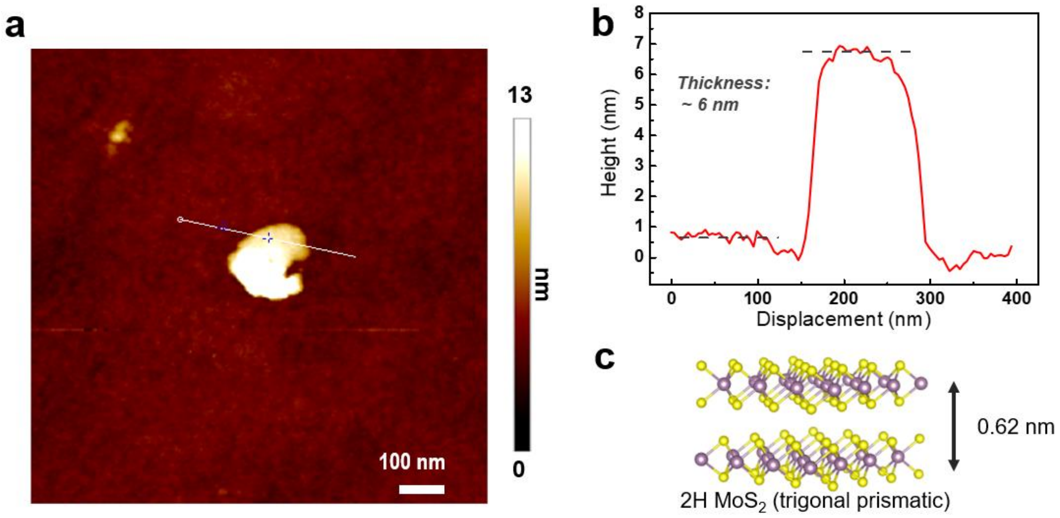

Figure S7. Characterization of the thickness of MoS_2 nanoflakes. (a) AFM image of a single flake of MoS_2. (b) Cross-section profile extracted from the AFM image. (c) Layer-to-layer distance of MoS_2 nanoflakes.

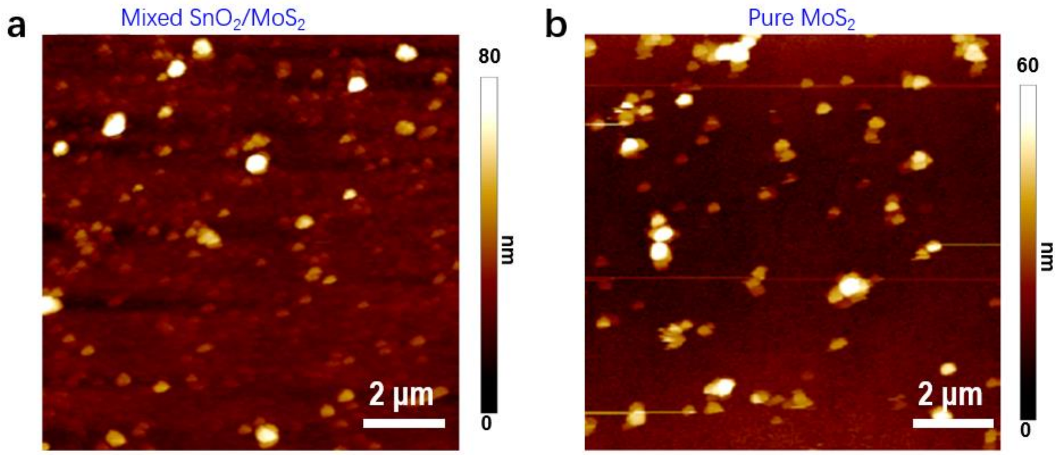

Figure S8. AFM characterization. (a) AFM image of the thin film fabricated using mixture of SnO_2 nanoparticles and MoS_2 nanoflakes. The aggregates (up to 600 nm) distributed evenly on the surface correspond to MoS_2 nanoflakes, while the granular texture on the surface correspond to the closely packed SnO_2 nanoparticles. (b) AFM image of the thin film fabricated using pure MoS_2 nanoflakes for comparison purpose, which reveals the presence of large-size MoS_2 nanoflakes and the absence of continuously packed SnO_2 nanoparticles.

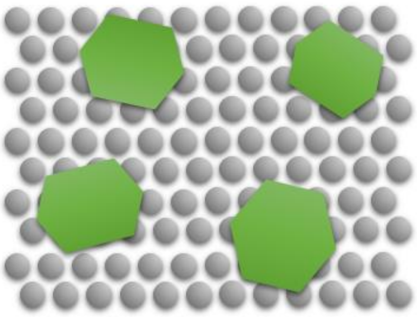

Figure S9. Schematic illustration (not to scale) of the structural morphology of the semiconducting film fabricated using solution-processing SnO_2 nanoparticle/MoS_2 nanoflake mixture. Based on the SEM and AFM results, it can be inferred that the large-size nanoflakes (hundreds of nm) were evenly distributed as aggregates, while the small-size nanoparticles (less than 10 nm) were closely packed as a continuous film with nanometer textures.

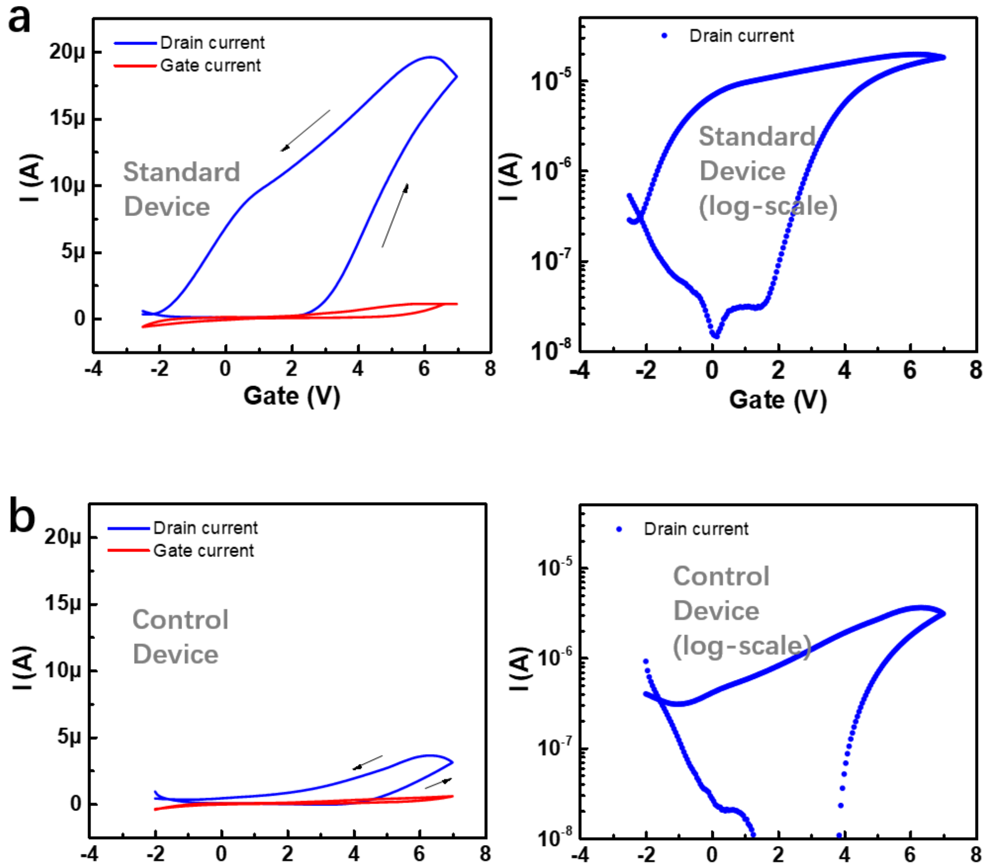

Figure S10. Comparison of transistor characteristics of a standard device and a control device under bias voltage V_{ds} = 0.75 V. (a) Transfer curve (linear scale and log scale) of the synaptic transistor fabricated as a standard device using SnO_2-nanoparticle/MoS_2-nanoflake mixture. This standard device shows large anticlockwise hysteresis (window of 4.5 V at 1 μA), a low threshold voltage (V_{th} = 2 V), a high on/off ratio (1130), a low leakage current (ratio of drain current to leakage current I_d/I_g = 19), and decent carrier mobility (μ = 0.3 cm^2/V/s). (b) Transfer curve (linear scale and log scale) of the synaptic transistor fabricated as a control device using pure SnO_2 nanoparticles. Compared with the standard device, this control device shows narrower hysteresis window (2.8 V at 1 μA), higher threshold voltage (V_{th} = 3.5 V), lower on/off ratio (112), larger leakage current (I_d/I_g = 5.5), and reduced carrier mobility (μ = 0.06 cm^2/V/s). Hence, the standard device fabricated using SnO_2/MoS_2 exhibit better device performance than the control device prepared using pure SnO_2; this comparison implies that the MoS_2 nanoflakes in the device channel can promote charge trapping and increase carrier mobility and electrostatic modulation.

Notes:

Carrier mobility μ in the linear regime, given $|V_g - V_{th}| > |V_{ds}|$, was calculated from the transfer curve by using:

$$\mu = \frac{L}{W C_i V_{ds}} \cdot \left(\frac{\partial I_d}{\partial V_g}\right)$$

where I_d and V_g are respectively drain current and gate voltage in the saturated regime, W is channel width, L is channel length, V_{ds} is the bias voltage, V_{th} is the threshold voltage, and C_i is the gate-channel capacitance per unit area.[1]

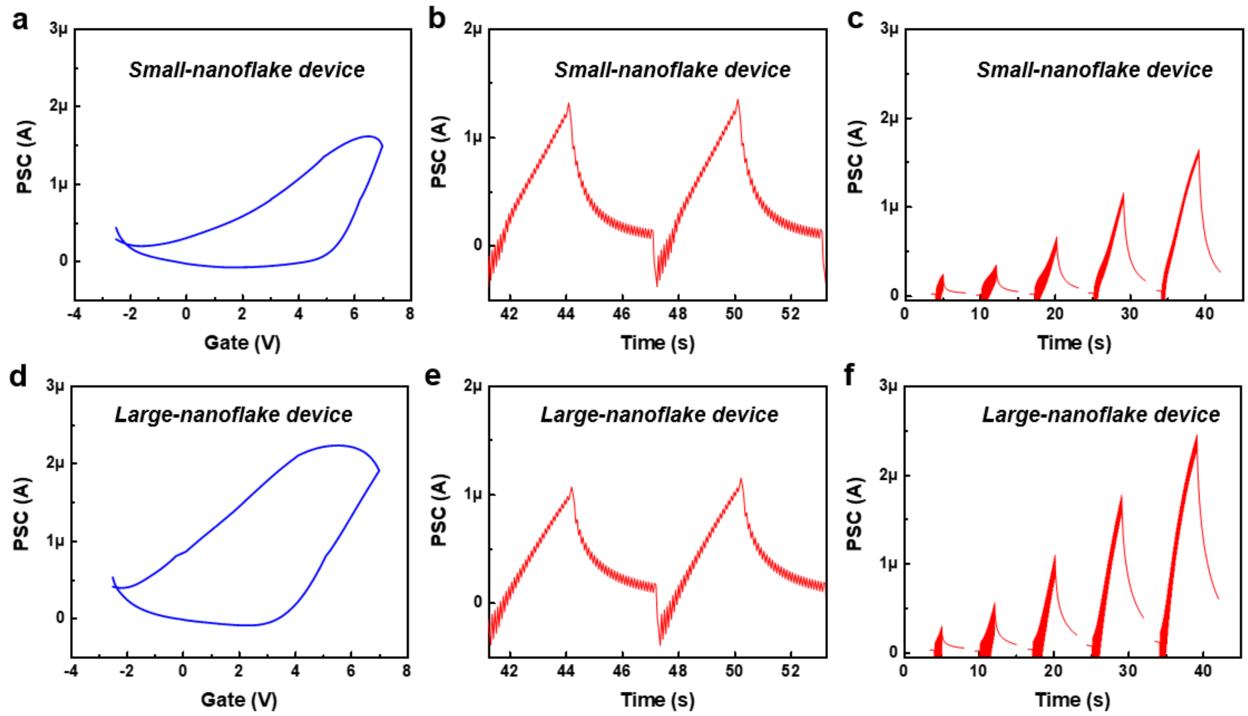

Figure S11. Influence of nanoflake size on the device performance. (a–c) Transfer curve (a), synaptic potentiation behavior (b), and spike-number dependent plasticity (c) of the device fabricated using small-size nanoflakes (100–300 nm). (d–f) Transfer curve (d), synaptic potentiation behavior (e), and spike-number dependent plasticity (f) of the device fabricated using large-size nanoflakes (400–700 nm).

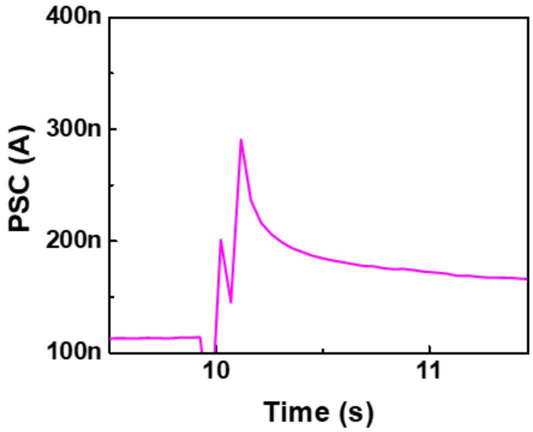

Figure S12. Paired-pulse facilitation (PPF) effect of the synaptic device. Postsynaptic current (PSC) was measured by applying two pulses (7 V, 50 ms) with an inter-spike interval of 100 ms; facilitation effect was observed.

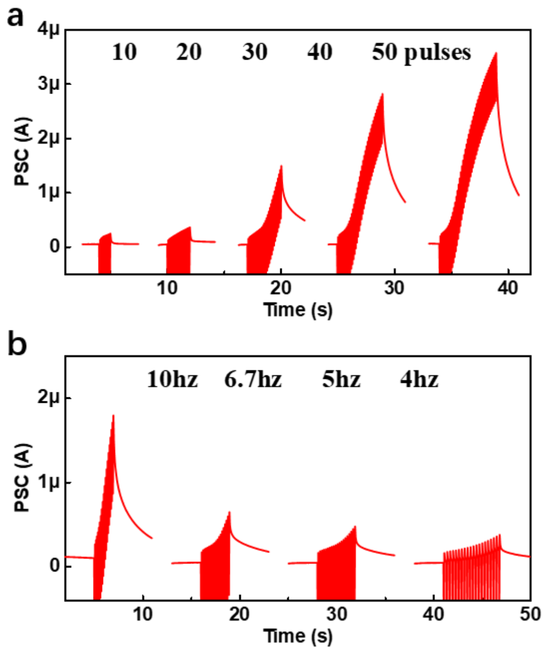

Figure S13 Performance of the control device fabricated using pure SnO_2 nanoparticles. (a) Spike-number dependent plasticity of the device under the stimuli of spike trains with various spike numbers. (b) Spike-rate dependent plasticity of the device under the stimuli of spike trains with various frequency. In terms of synaptic characteristics, the control device (SnO_2 channel) shows reduced linearity and increased leakage current compared with the standard device (SnO_2/MoS_2 channel). Thus it can be inferred that the introduction of MoS_2 in the channel can improve the synaptic behaviors of the device towards neuromorphic applications.

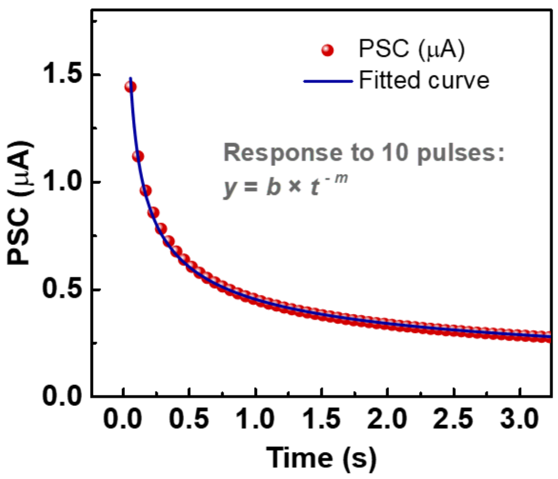

Figure S14. Decay of the postsynaptic current from the synaptic device after the stimuli of voltage spikes (7 V, 50 ms, 10 pulses, 10 Hz) is stopped. This current-retention process is analogous to memory retention in the brain, and the decay of the postsynaptic current is similar to that of forgetting curve, which shows how information is lost over time without memory rehearsal. This decay curve is well fitted to a widely used forgetting curve, defined as: $y = b \times t^{-m}$, where y is memory retention (y is postsynaptic current in this case), t is time ($t = 0$ when the stimuli stops), b and m ($b = 0.45$, $m = 0.42$; m is regarded as the time constant) are fitted parameters. Time constant of this decay curve (0.42 s) is within the range of human sensory memory (0.2 – 3 s).

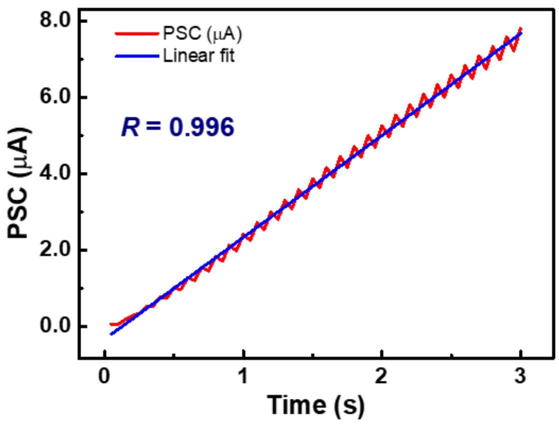

Figure S15. Typical potentiation behavior of the synaptic device under the stimuli of consecutive spike trains. The postsynaptic current (PSC) curve shows a high correlation coefficient ($R = 0.996$) of linear fit, indicating the linear potentiation property of the device.

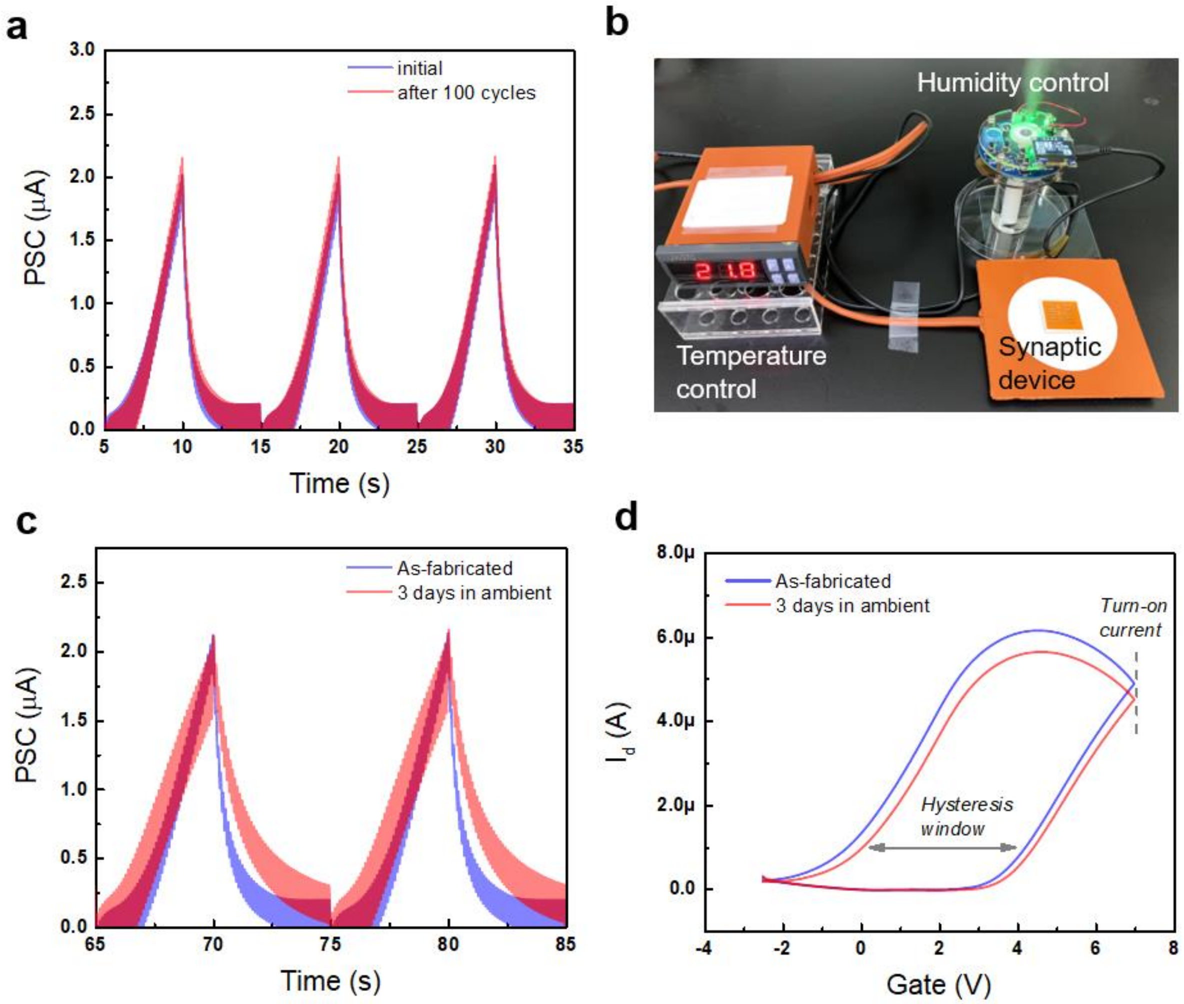

Figure S16. Operation reliability and environment stability of the synaptic device. (a) Postsynaptic current of the device before and after 100 cycles of potentiation/depression spikes. (b) A custom-built equipment for controlling both temperature and humidity. (c) The synaptic potentiation/depression behavior of the device measured before and after 3-day storage in ambient condition. (d) The transfer curve of the device measured before and after 3-day storage in ambient condition.

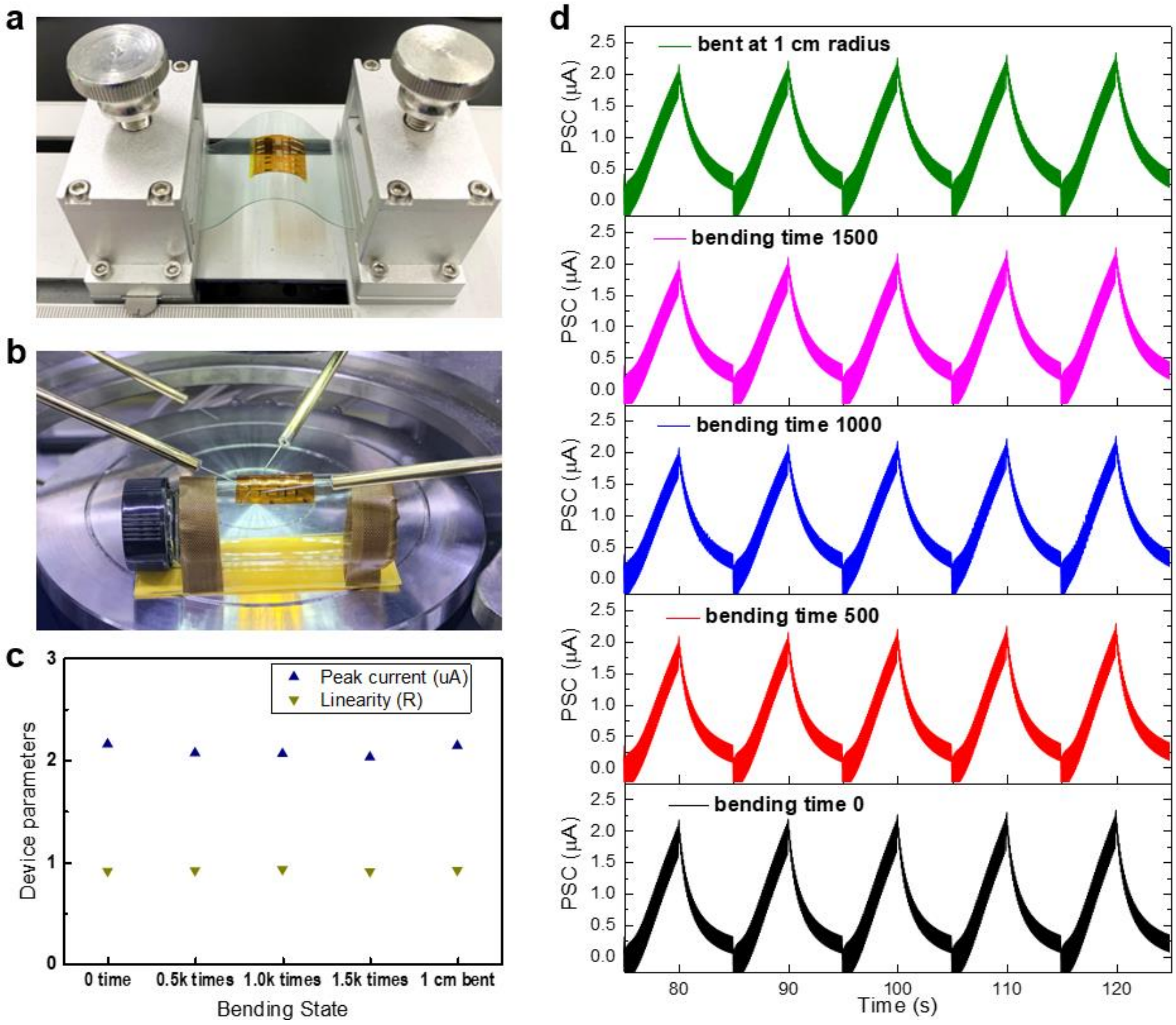

Figure S17. Flexible characteristics of the synaptic device. (a) Setup for cyclic bending test of the device (repeated bending for 1500 times). (b) Setup for static bending test of the device (fixed bending radius of 1 cm). (c) Device parameters evaluated at different bending states (0 bending time, 500 bending times, 1000 bending times, 1500 bending times, bent at 1 cm radius). (d) Synaptic characteristics measured at different bending states.

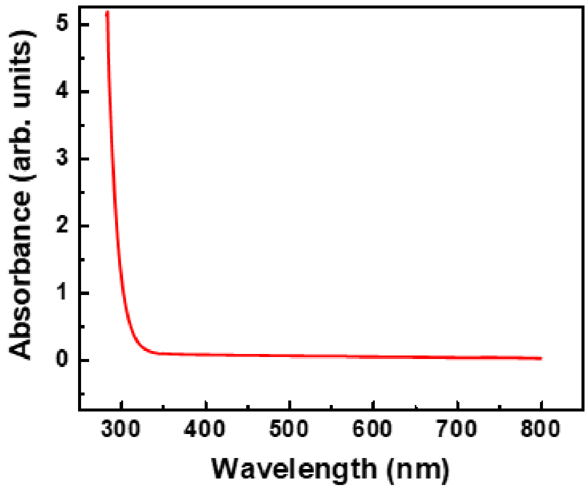

Figure S18. UV-visible absorption spectra of the SnO_2 nanoparticles showing the UV-sensitive property.

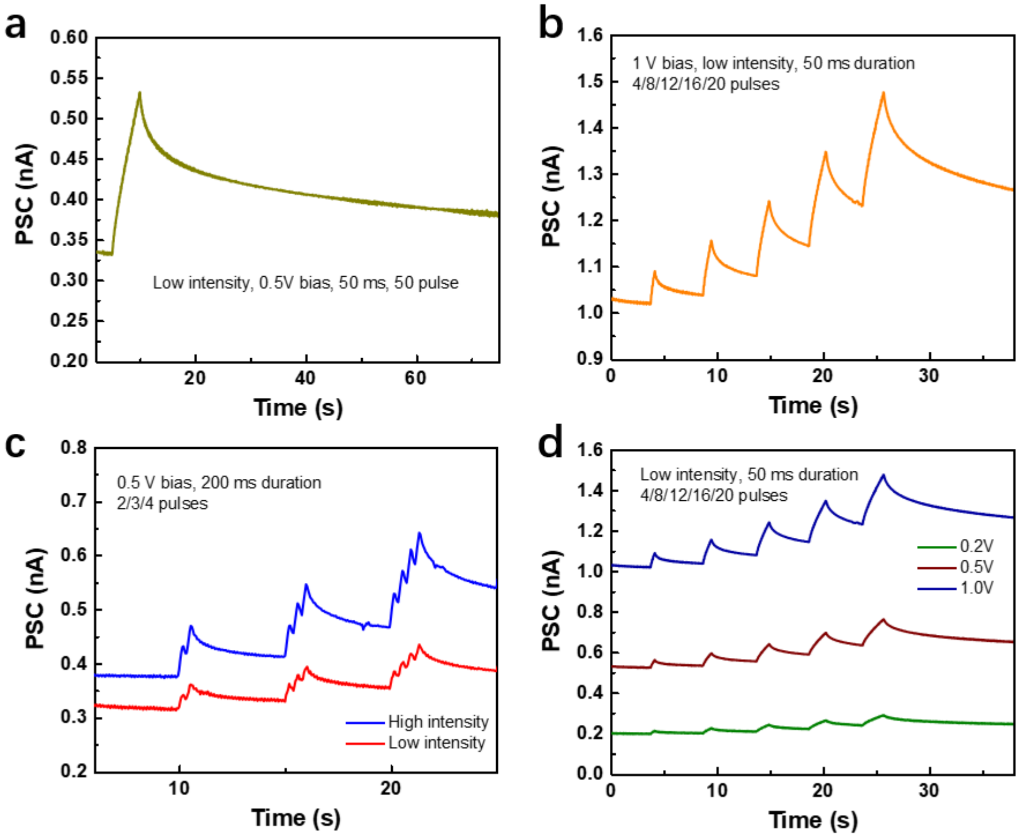

Figure S19. Response of synaptic device under the stimuli of 250-nm optical pulses. (a) Postsynaptic current under application of 50 optical pulses (light intensity 8 μW/cm^2, bias 0.5 V, spike duration 50 ms). This memory retention curve shows behavior of short-term memory (time constant of 57 s, derived by fitting to a memory-loss curve). (b) Postsynaptic current under application of optical pulses with various pulse number (pulse number 4, 8, 12, 16, or 20; light intensity 8 μW/cm^2; bias 1.0 V; spike duration 50 ms). (c) Postsynaptic current under application of optical pulses with different light intensity (light intensity 8 or 16 μW/cm^2; bias 0.5 V; spike duration 200 ms). (d) Postsynaptic current in response to optical pulses under different bias voltage (bias voltage 0.2, 0.5, or 1.0 V; light intensity 8 μW/cm^2; spike duration 50 ms). These results, together with the observations in Figure 2, demonstrate that the plasticity of the synaptic device can be effectively modulated by both electrical and optical pulses.

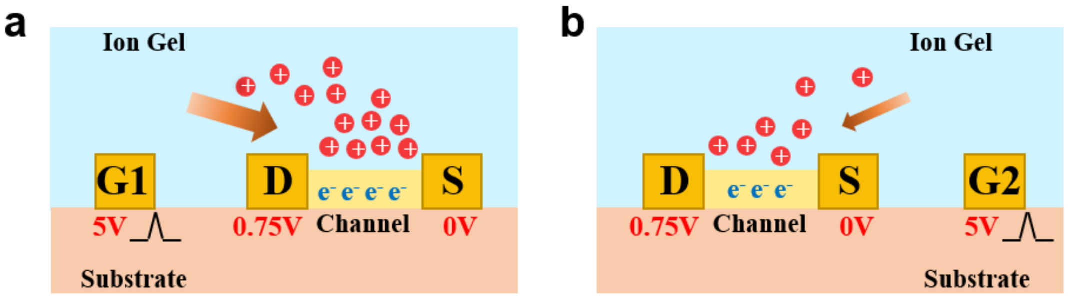

Figure S20. Schematic illustration of the different modulatory effects of major gate (denoted as Gate1) and minor gate (denoted as Gate2) in the synaptic transistor, under bias voltage of 0.75 V. (a) When positive spikes (5V) are applied to Gate1, which is close to drain terminal (0.75 V), the electrostatic potentiation modulation effect is strong. (b) When positive spikes (5V) are applied to Gate2, which is close to source terminal (0 V), the electrostatic potentiation modulation effect is weak. Therefore, location of the two different gates in the synaptic transistor were appropriately designed so that they have different modulatory effect under positive spikes.

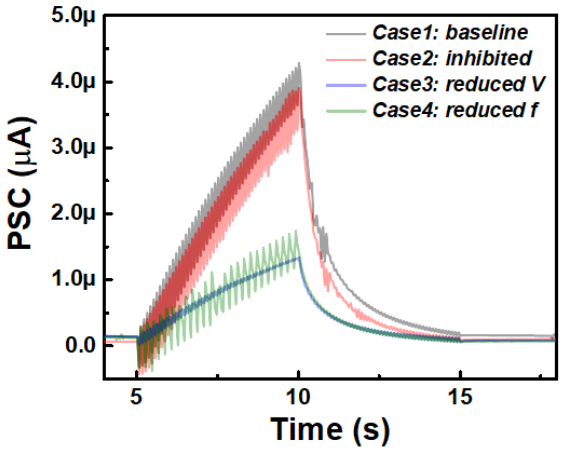

Figure S21. Modulation of perceptual weight of the synaptic device. In Case1, postsynaptic current of the device under positive voltage spikes (+7 V, 10 Hz) is measured and defined as the baseline. In Case2, synaptic plasticity of the device is initially depressed by applying negative spikes (−2.5 V, 10 Hz), and then the postsynaptic signal under positive spikes (+7 V, 10 Hz) is measured. In Case3, positive voltage spikes with reduced amplitude (+3.5 V, 10 Hz) are applied. In Case4, positive voltage spikes with reduced frequency (+7 V, 5 Hz) are applied. In principle, device properties including history-dependent plasticity and spike-dependent plasticity can be utilized for modulating the perceptual weight.

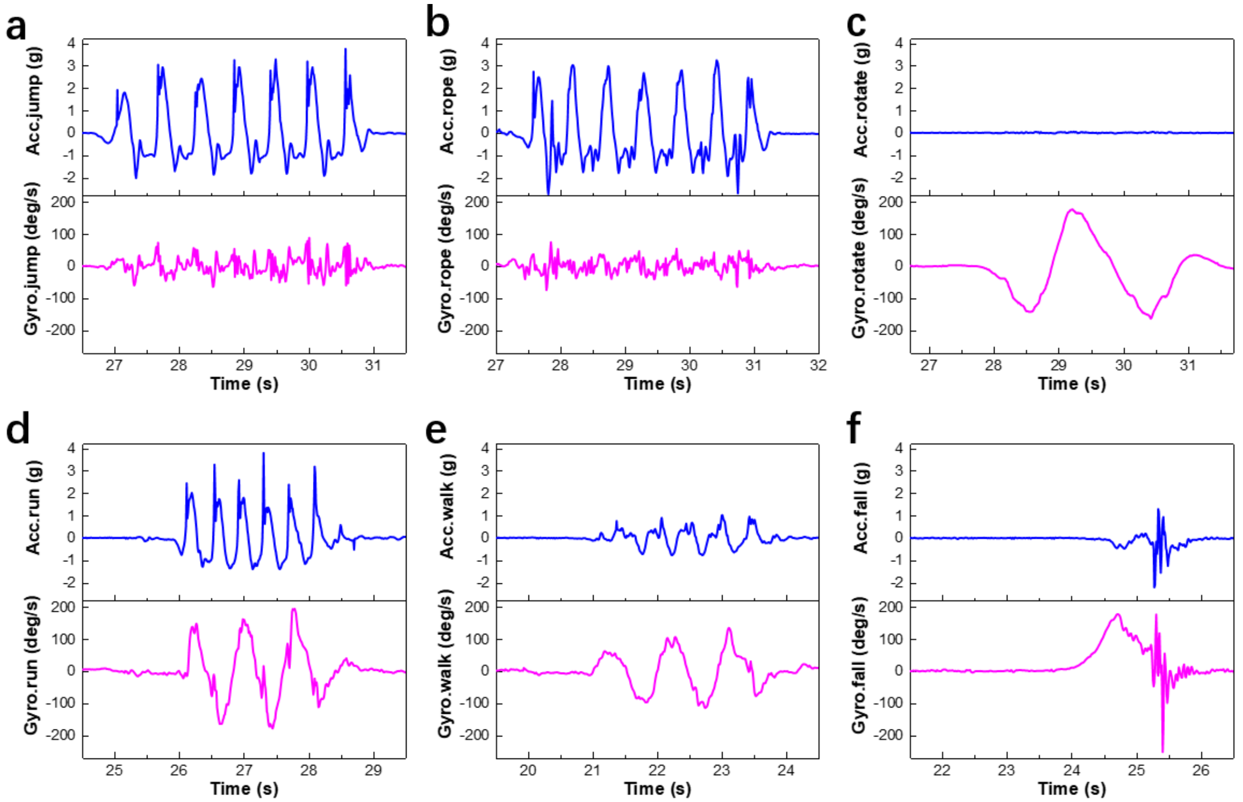

Figure S22. Typical bimodal motion signals of human activities. Acceleration (Acc) and gyroscopic (Gyro) signals corresponding to six different human activities: (a) jumping, (b) rope skipping, (c) torso rotation, (d) running, (e) race walking, and (f) falling.

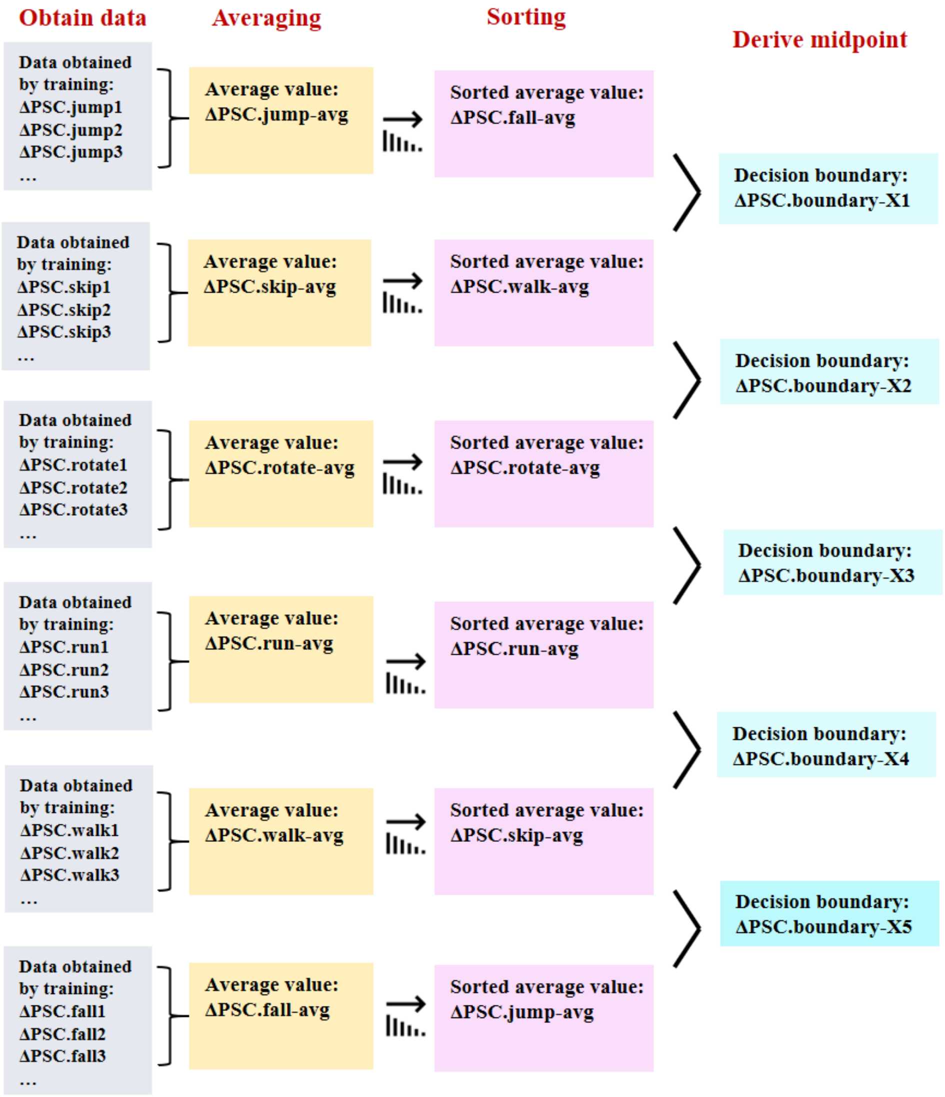

Figure S23. Flow chart showing deriving the decision boundaries (ΔPSC) for classifying human activity type in a training process. First, all the data point of ΔPSC obtained from a training process are grouped as six data clusters corresponding to the six motion types. Second, average values of the six data clusters are derived. Third, the obtained average values are sorted. Finally, decision boundaries are derived as the midpoint of two neighboring values, yielding five decision boundaries which can classify all the six motion types. Decision boundaries of mean firing rates (f_{acc}, f_{gyro}) are derived in the same method.

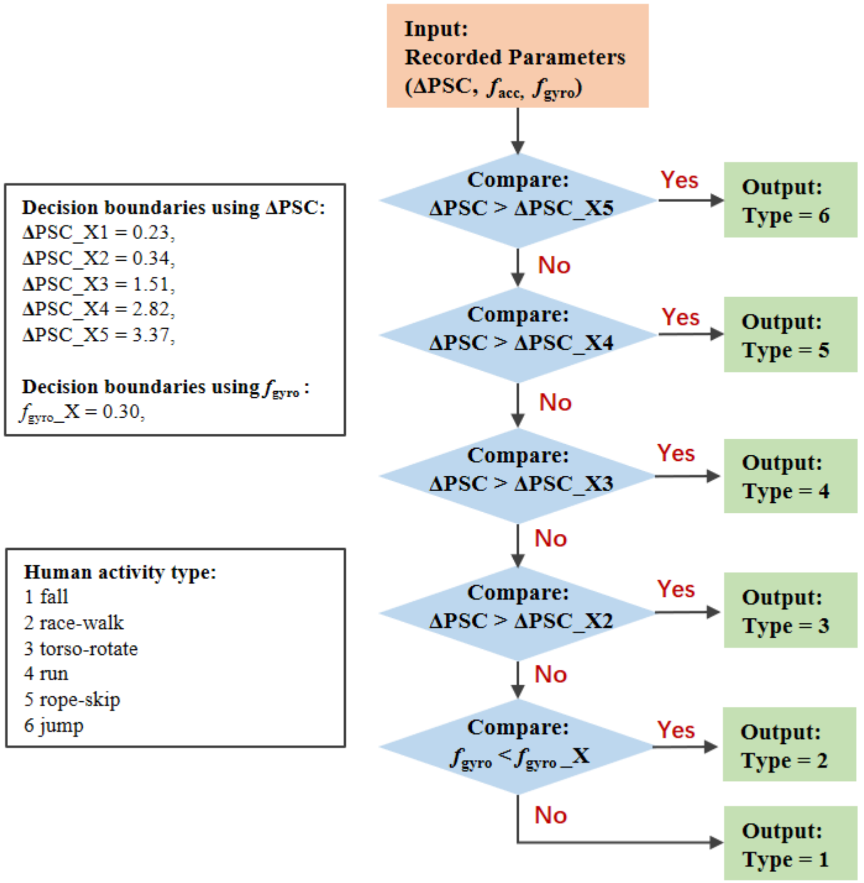

Figure S24. Flow chart showing the comparison operations for recognizing human activity in a testing process. The type of human activity is classified by simply comparing the hardware outputs (ΔPSC, f_{acc}, f_{gyro}) with their decision boundaries. Some decision boundaries for ΔPSC (ΔPSC_X1, ΔPSC_X2) are close to each other, so additional decision boundaries for f_{gyro} (f_{gyro}_X) were introduced to improve the classification accuracy.

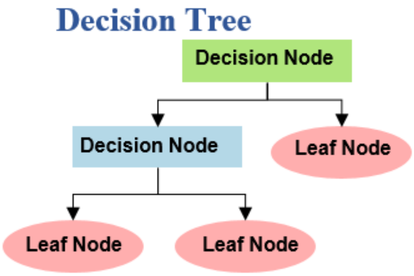

Figure S25. Illustration of the principle of the decision tree method used for recognition tasks. A decision node represents a comparison of hardware outputs with their decision boundaries; multi-level decision making can be implemented; each leaf node represents a prediction. In such way, classification can be achieved using simple operations of comparison.

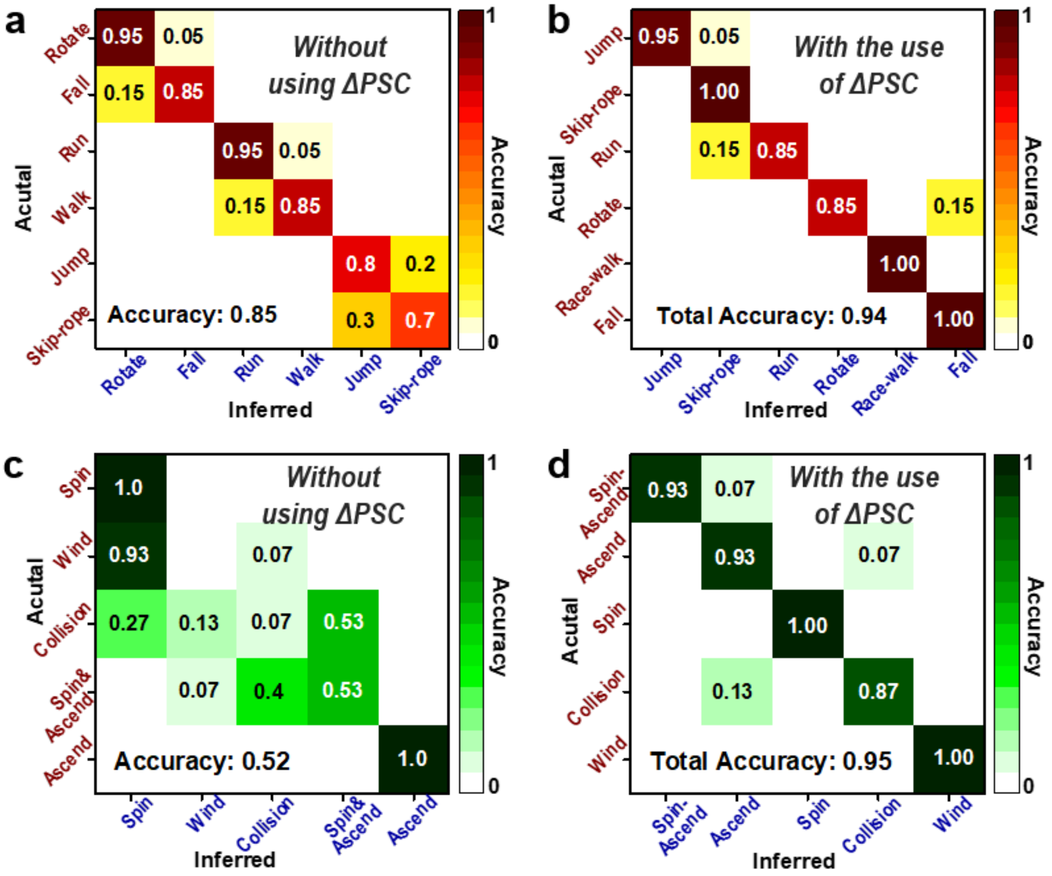

Figure S26. Introduction of postsynaptic current change (ΔPSC) for improving motion recognition accuracy. (a) Classification results without added feature of ΔPSC for human activity recognition task. (b) Classification results with added feature of ΔPSC for human activity recognition task. (c) Classification results without added feature of ΔPSC for drone flight mode recognition task. (d) Classification results with added feature of ΔPSC for drone flight mode recognition task. By adding postsynaptic current change (ΔPSC) as classification criterion, the total accuracy for both human-motion and drone-motion recognition tasks are increased.

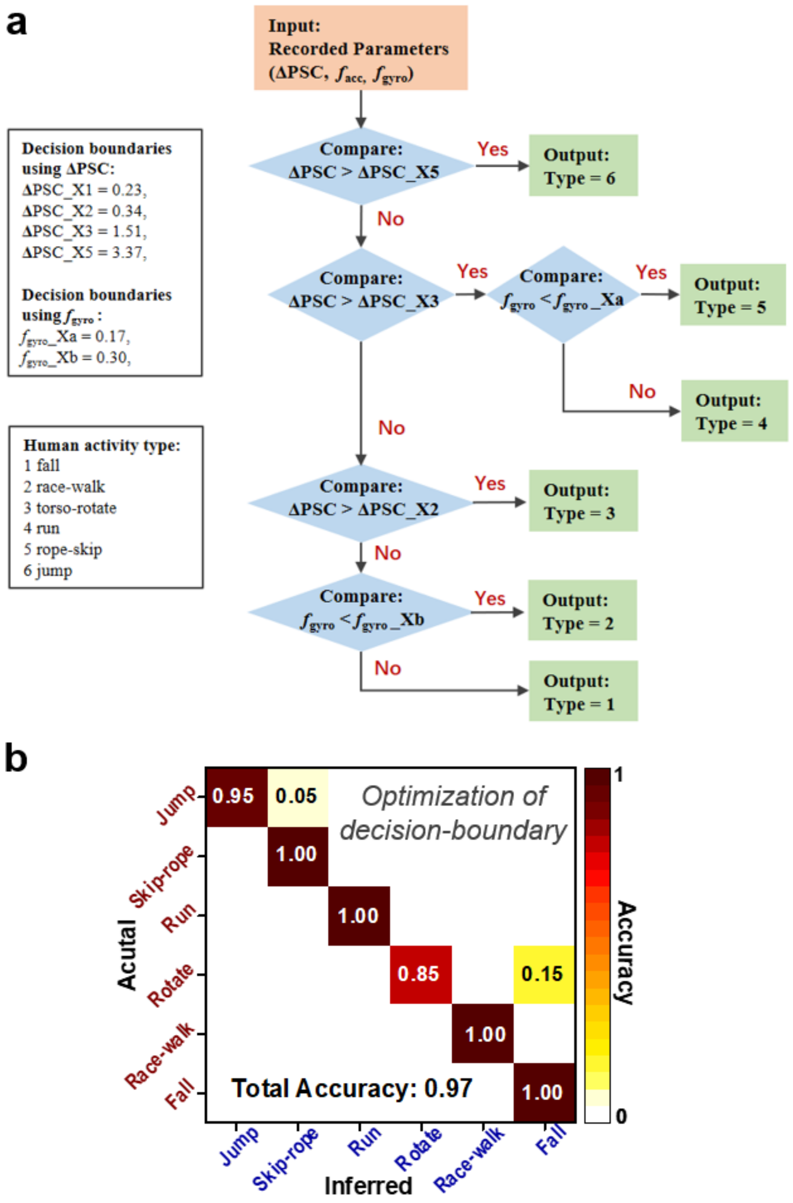

Figure S27. Further improvement of the recognition accuracy of human motion. (a) Optimization of the decision boundary in the decision-tree method. (b) Classification results for human activity recognition task after decision-boundary optimization.

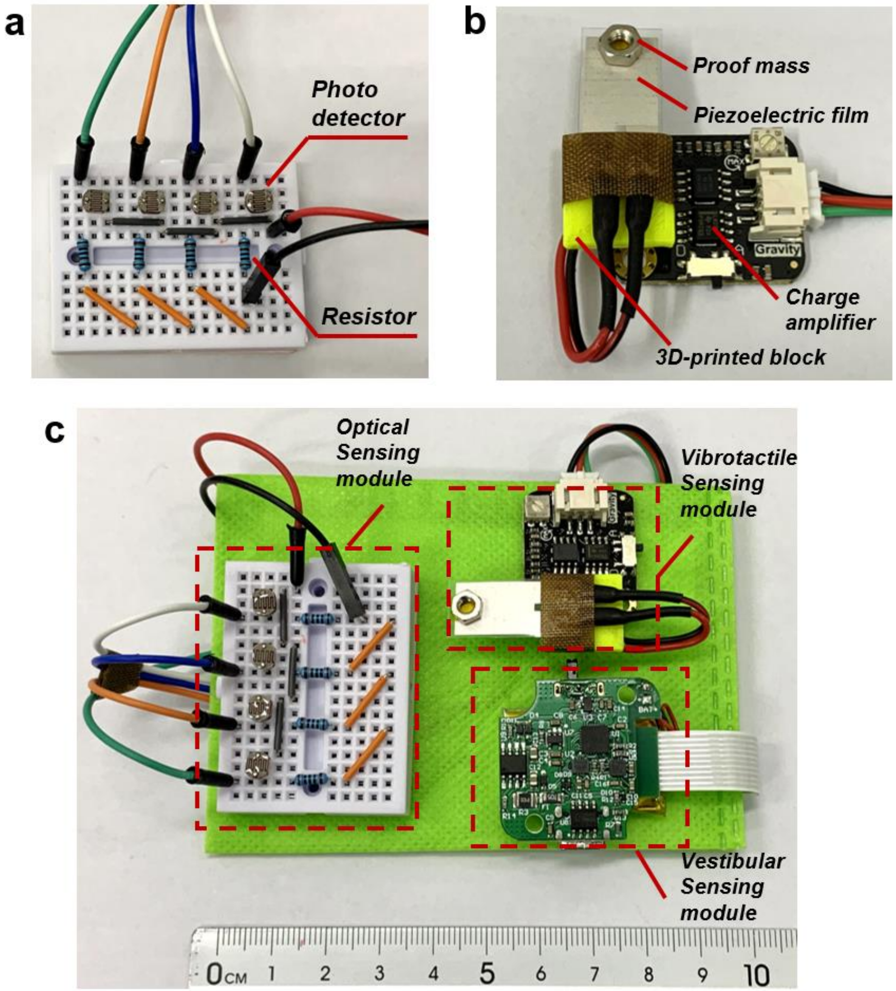

Figure S28. Development of different sensing modules in the neuromorphic motion-cognition system. (a) Optical sensing module built from photo detector array. (b) Vibrotactile sensing module built from PVDF piezoelectric film. (c) Sensing unit comprising the optical, the vibrotactile, and the vestibular sensing modules (all mounted onto a flexible fabric) for obtaining visual, tactile, and vestibular sensory cues.

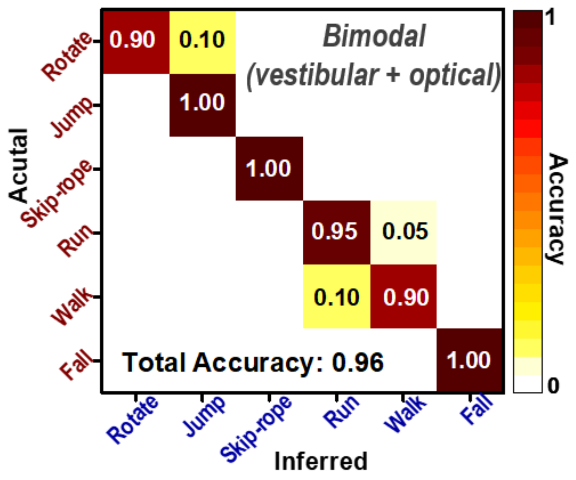

Figure S29. Classification result for human activity recognition task when vestibular and optical cues are integrated.

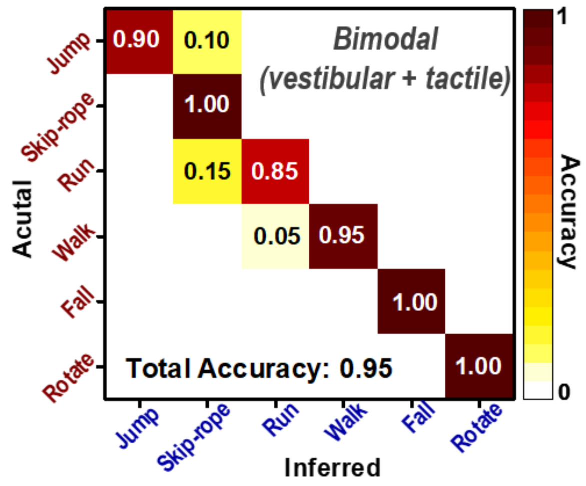

Figure S30. Classification result for human activity recognition task when vestibular and vibrotactile cues are integrated.

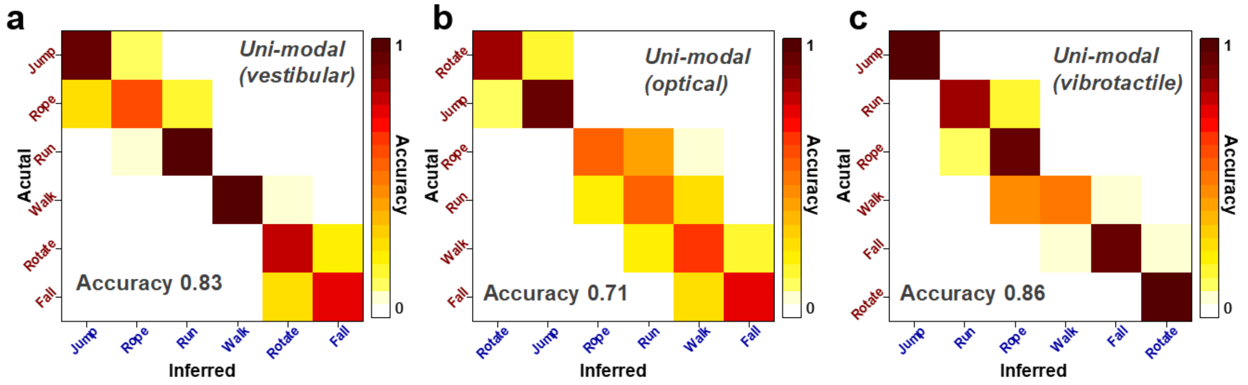

Figure S31. Investigation on motion recognition performance when each sensory cue is segregated under unimodal sensory condition. (a) Classification result for human activity recognition task when only vestibular cue is obtained. (b) Classification result for human activity recognition task when only optical cue is obtained. (c) Classification result for human activity recognition task when only vibrotactile cue is obtained.

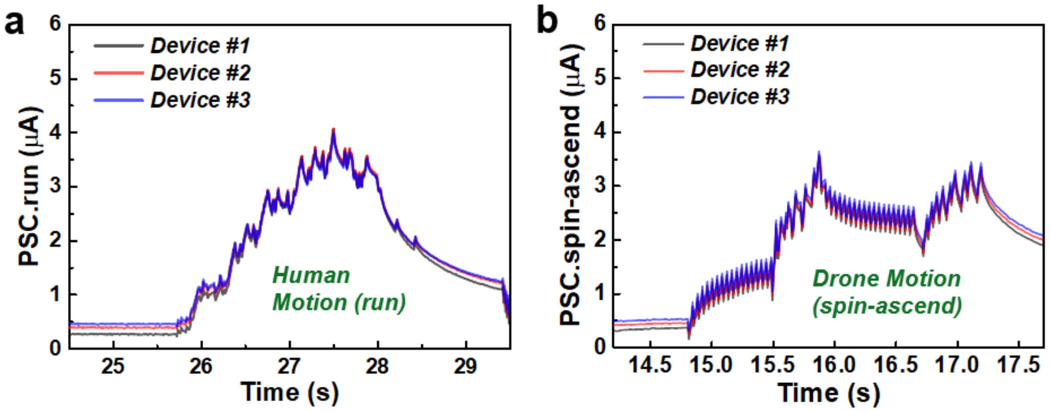

Figure S32. Investigation on the device-to-device variation of synaptic response. (a) Postsynaptic current of three different devices (Device #1, #2, #3) in response to human motion signal (run motion). (b) Postsynaptic current of three different devices (Device #1, #2, #3) in response to drone motion signal (spin-ascend motion).

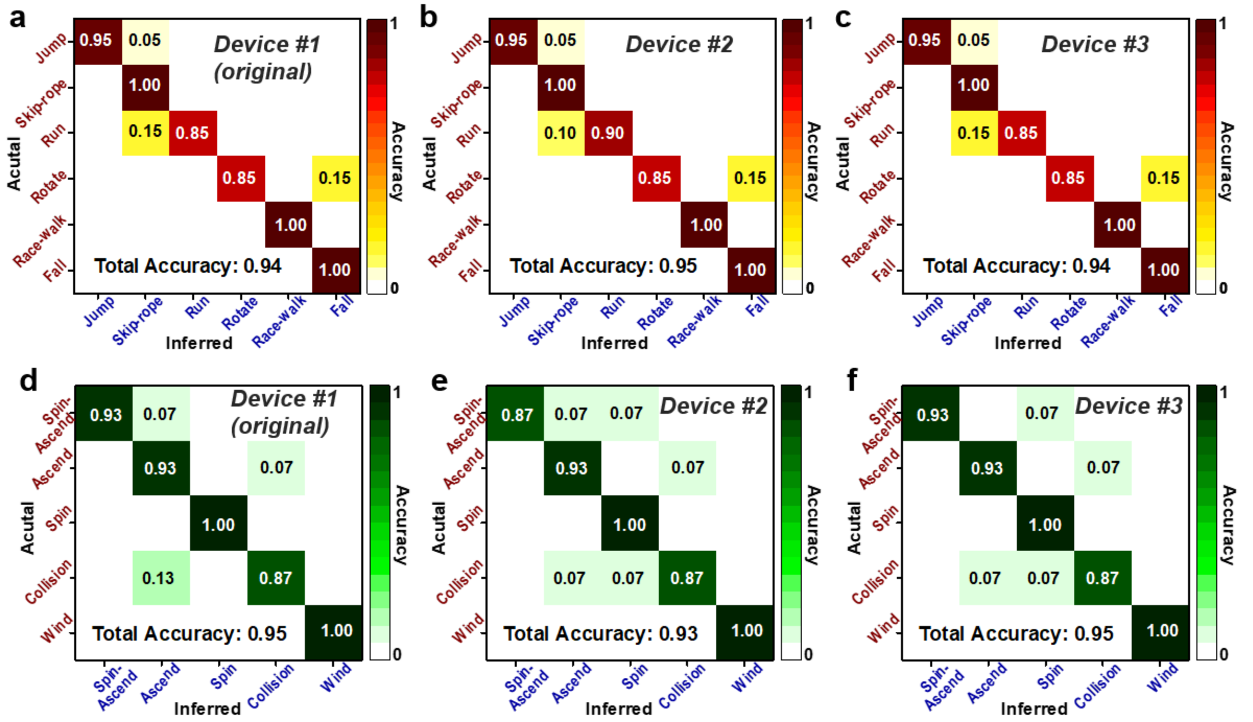

Figure S33. Investigation on the device-to-device variation of motion-recognition performance. (a-c) Classification results of human motion types using Device #1 (a), Device #2 (b), and Device #3 (c). (d-f) Classification results of drone flight modes using Device #1 (d), Device #2 (e), and Device #3 (f).

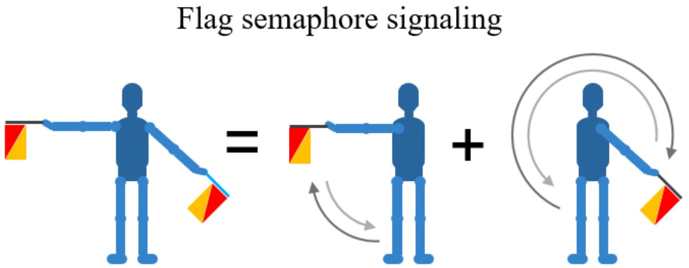

Figure S34. Illustration of the flag semaphore signaling process involving the waving of hand-held flag. For simplicity, the two-flag waving motion was divided into sequential motions of one-flag waving, so that the signaling was performed using one arm and meanwhile the arm motion was detected using a single IMU sensor.

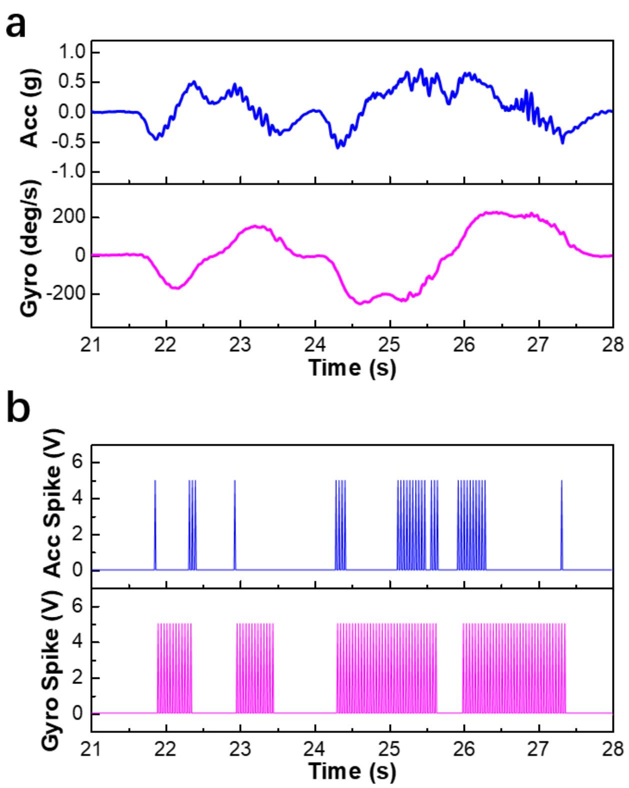

Figure S35. Typical bimodal motion signals of flag semaphore. (a) The acquired signals of acceleration (Acc) and gyroscopic angular speed (Gyro). (b) The encoded spike trains corresponding to the two signals in (a). Signaling using semaphore involves flag-waving motions that generate temporally correlated signals of rotational inertia and angular speed. This case presents flag semaphore of letter "S".

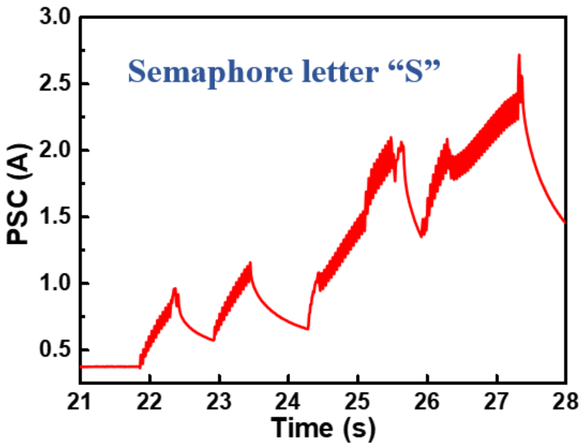

Figure S36. The postsynaptic current of the device when semaphore flag of letter "S" was signaled by waving a flag clockwise for 90°, −90°, 315° and −315° with a short pause in between.

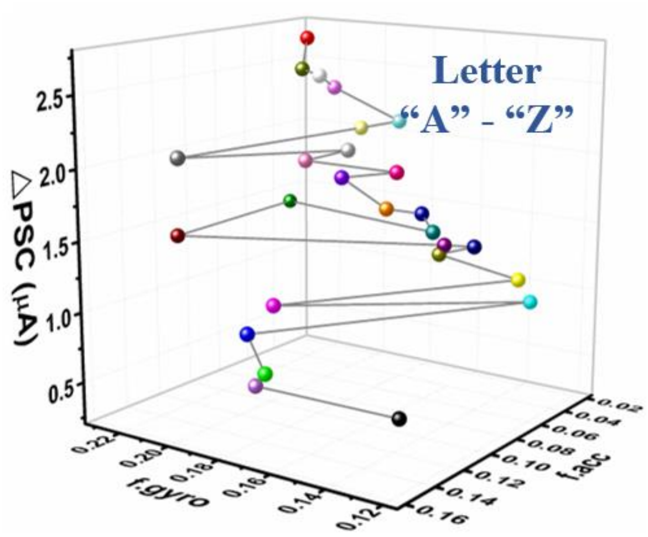

Figure S37. 3D visualization of the hardware outputs (f_{acc}, f_{gyro}, ΔPSC; typical values) for flag semaphore classification, wherein the 26 data points in different colors correspond to the alphabet letters.

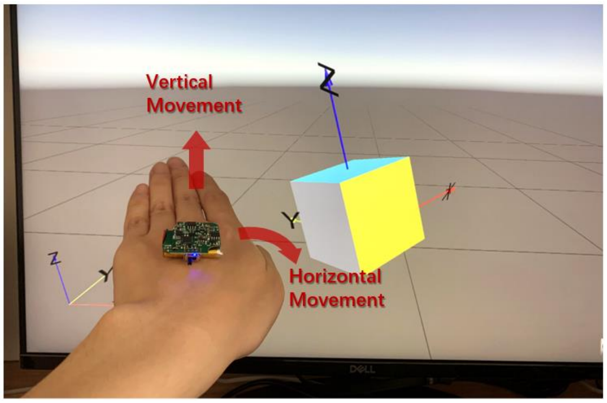

Figure S38. Demonstration of hand gesture classification. A human subject wearing the IMU sensor on back of the palm performed simple hand gestures. The bimodal motion signals were wirelessly transmitted to the synaptic electronics and a monitor, for processing and display.

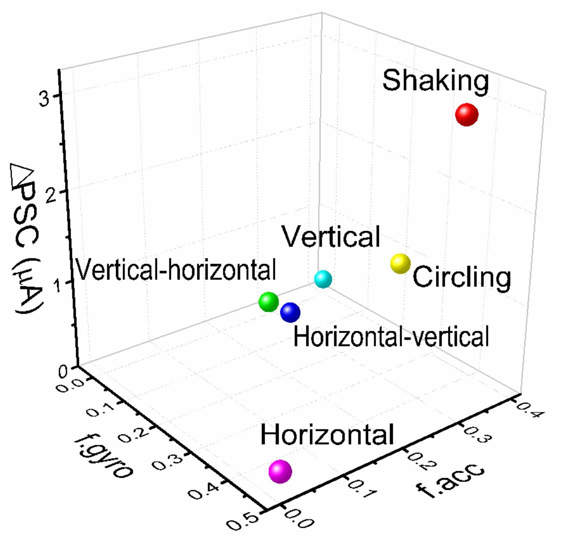

Figure S39. 3D visualization of hardware outputs (f_{acc}, f_{gyro}, ΔPSC; typical values) for hand gesture classification of six hand gestures: shaking, circling, horizontal, vertical, vertical-horizontal, and horizontal-vertical movements. All hand gestures can be well distinguished.

Notes:

Shaking: shaking hand continuously.

Circling: moving hand in circle, regardless of anticlockwise or clockwise.

Horizontal: moving hand horizontally in left or right direction.

Vertical: moving hand vertically in upward or downward direction.

Vertical-horizontal: first moving hand vertically, then horizontally.

Horizontal-vertical: first moving hand horizontally, then vertically.

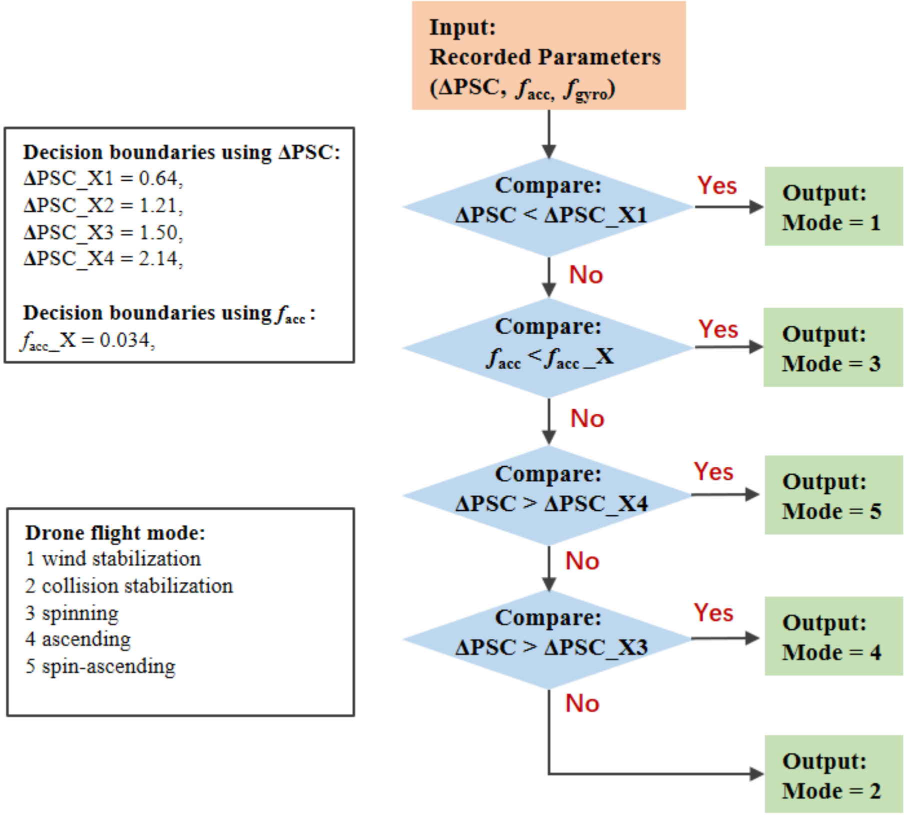

Figure S40. Flow chart showing the comparison operations for recognizing drone flight mode. Drone flight mode is classified by simply comparing the hardware outputs (ΔPSC, f_{acc}, f_{gyro}) with their decision boundaries. Some decision boundaries for ΔPSC (ΔPSC_X3 and ΔPSC_X2) are close to each other, so additional decision boundary for f_{acc} (f_{acc}_X) were introduced to improve the classification accuracy.

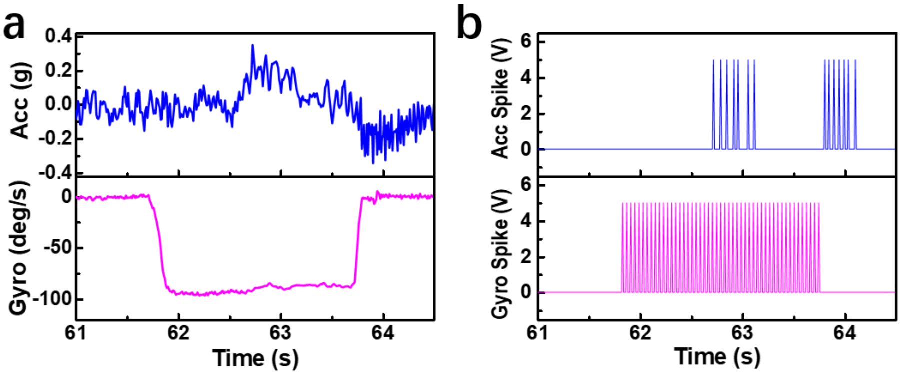

Figure S41. Typical bimodal motion signals of drone flight. (a) Acceleration (Acc) and gyroscopic angular speed (Gyro) signals acquired during the spin-ascending operation. (b) The encoded spike trains corresponding to the bimodal signals in (a) during the spin-ascending operation.

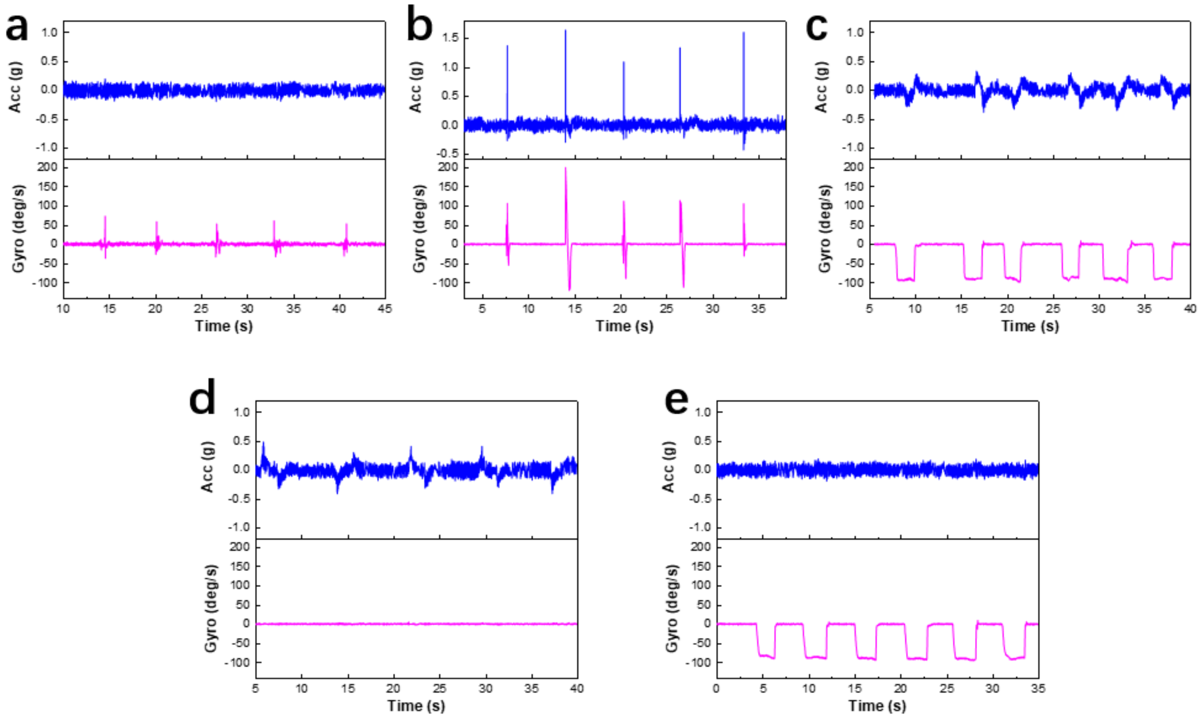

Figure S42. Typical bimodal motion signals of quadrotor drone operated in different flight mode. Acceleration (Acc) and gyroscopic angular speed (Gyro) bimodal signals corresponding to five different drone flight modes of (a) wind stabilization, (b) collision stabilization, (c) spinning, (d) ascending (or descending), and (e) spin-ascending (or spin-descending) are presented.

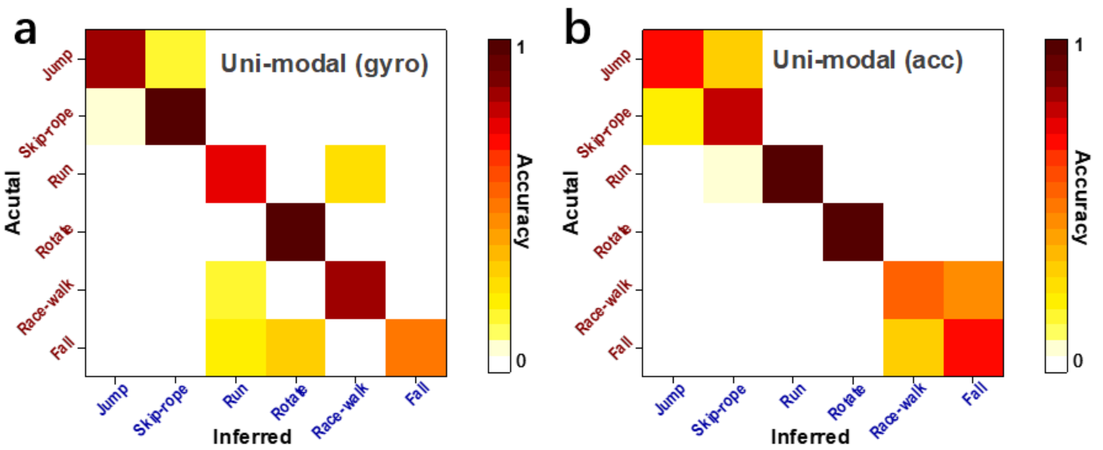

Figure S43. Results of human activity recognition in uni-modal conditions. (a) Confusion matrix of human-activity recognition performed using angular speed signal of gyroscope (Gyro) as uni-modal sensory input. (b) Confusion matrix of human activity recognition performed using acceleration signal of accelerometer (Acc) as uni-modal sensory input.

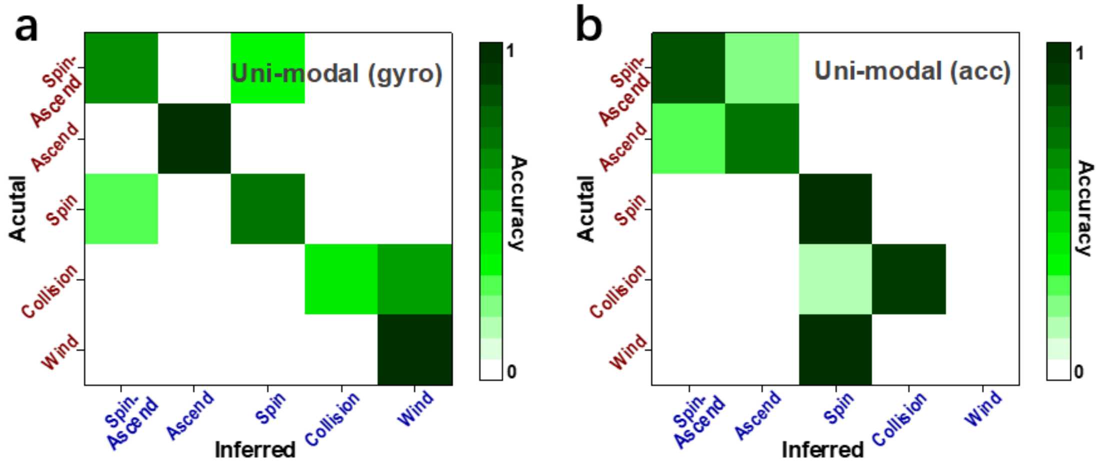

Figure S44. Results of drone flight-mode recognition in uni-modal conditions. (a) Confusion matrix of drone flight mode recognition performed using angular speed signal of gyroscope (Gyro) as uni-modal sensory input. (b) Confusion matrix of drone flight mode recognition performed using acceleration signal (Acc) of accelerometer as uni-modal sensory input.

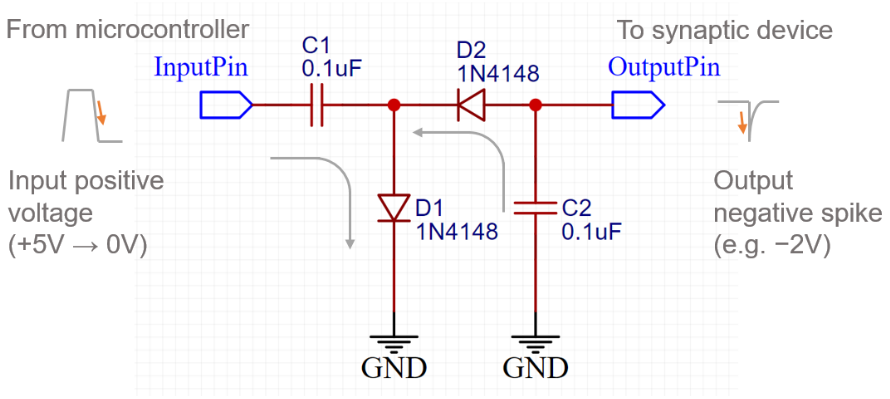

Figure S45. The capacitor-diode circuit for resetting the synaptic device.

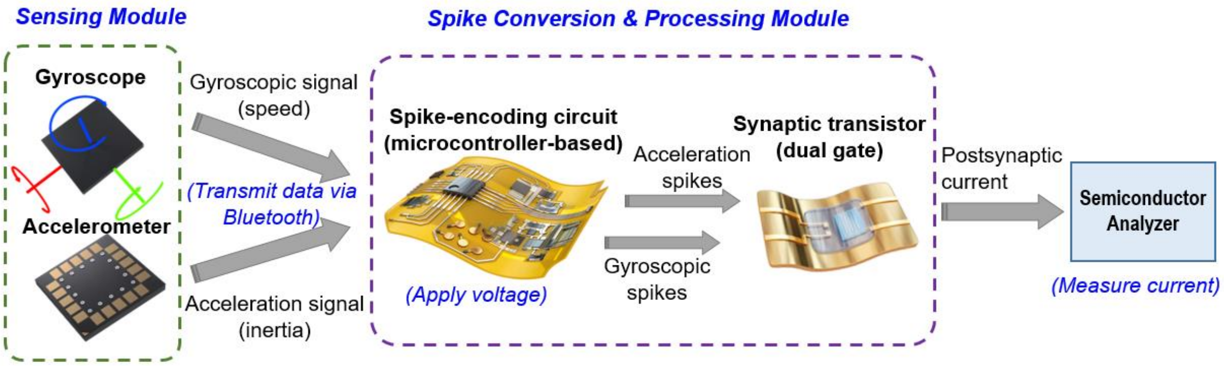

Figure S46. Schematic illustration of the signal flow in the neuromorphic motion-cognition system.

Table S1. Comparison of two synaptic devices fabricated using nanoflakes with different size.

**Device No.**	**Hysteresis window**	**Threshold voltage**	**Leakage current ratio (I_d/I_g)**	**Turn-on current**	**Signal-to-Noise Ratio**
**#1 Small-nanoflake device**	*2.7 V*	*4.3 V*	*16.7*	*1.48 μA*	*22.5*
**#2 Large-nanoflake device**	*4.9 V*	*3.0 V*	*13.0*	*1.90 μA*	*12.2*

Table S2. Comparison of recently reported synaptic devices fabricated using solution-processable 2D materials. LB: Langmuir-Blodgett method; LIF: leaky integrate and fire; PPF: paired-pulse facilitation.

**Type of synaptic device**	**Semiconducting materials**	**Fabrication technique**	**Device characteristics**	**Synaptic Functions**
Hetero-structure memristive device [2]	MoS_2 nanoflake (MoO_x surface)	Wet coating (LB), oxidation	Bipolar resistive switching; flexible	Memory retention
Memristive device [3]	TiO_x nanosheet	Wet coating (LB), UV exposure	Volatile resistive switching; flexible	LIF behavior
Memtransistor [4]	MoS_2 nanosheet (sulfurized)	Spray coating, sulfurization	Bipolar resistive switching	Spike-dependent plasticity
Multi-layer memristive device [5]	WS_2 nanosheet, ZrO_2 thin film	Spin coating, sputtering	Bipolar resistive switching	Spike-dependent plasticity; PPF effect
Memristive device [6]	WS_2 nanosheet,	Drop casting	Bipolar resistive switching	Spike-dependent plasticity; PPF effect
Synaptic transistor (this work)	MoS_2 nanoflake, SnO_2 nanoparticle	Spin coating	Electrical/optical gating; multiple input; spatiotemporal integration; flexible	Spike-dependent plasticity; PPF effect; BCM effect; spatiotemporal recognition

Table S3. Modulation of synaptic weight by changing the characteristics of the applied voltage spikes.

**No. of Case**	**Case #1 (baseline)**	**Case #2 (inhibited)**	**Case #3 (reduced volt.)**	**Case #4 (reduced freq.)**
**Applied spikes**	+7 V, 10 Hz	−2.5 V, 10 Hz<br>+7 V, 10 Hz	+3.5 V, 10 Hz	+7 V, 5 Hz
**Peak postsynaptic current (μA)**	4.27	3.89	1.74	1.33
**Synaptic weight (μS)**	5.70	5.19	2.31	1.77

Table S4. Comparison between the conventional CMOS neuromorphic system and our synaptic-transistor based neuromorphic system.

**(Supplementary Ref.: 7 – 11)**	**Neuromorphic circuit using conventional CMOS devices**	**Neuromorphic circuit using flexible synaptic transistors**
**Number of device required for one synapse**	*Up to 10*	*1*
**Additional system clock**	*Required*	*Not required*
**Synaptic functions emulated**	*SNDP, SRDP, STDP, Leaky-Integrate-and-Fire neuron*	*SNDP, SRDP, Spatiotemporal integration, multi-sensory neuron*
**Energy consumption**	*High*	*Low*
**Mechanical flexibility**	*No (rigid)*	*Yes (flexible)*

Table S5. Correlation coefficient of the acquired bimodal signals (before encoding) compared with the correlation coefficient of the encoded spike trains (after encoding).

**Type of data**	**Correlation coefficient (acquired signals)**	**Correlation coefficient (encoded spikes)**
Metronome motion	−0.012	−0.015
Human motion	0.10	0.18
Drone motion	0.18	0.23

Note:
Correlation coefficient of two data sets were calculated using:

$$\rho_{XY} = \frac{Cov(X,Y)}{\sqrt{\mathrm{Var}(X) \cdot \mathrm{Var}(Y)}}$$

where X and Y represent paired data (x_i, y_i), and ρ_{xy} measures the strength of correlation. Theoretically, ρ_{xy} ranges from −1 to +1.

The correlation coefficients for metronome motion signals are negative due to temporal incongruence of the two signals generated from swing motion of metronome.

The correlation coefficients for human motion signals are derived from the data corresponding to typical motion of running.

The correlation coefficients for drone motion signals are derived from the data corresponding to typical motion of collision.

Table S6. Decision boundaries of synaptic response (ΔPSC in μA) for recognizing human activity types (jumping, rope skipping, torso rotation, running, race walking, and falling).

**Human activity**	**Decision boundaries (synaptic response, μA)**	
	**Low**	**High**
Falling	0.000	0.230
Race walking	0.230	0.344
Running	0.344	1.515
Torso rotation	1.515	2.821
Rope skipping	2.821	3.372
Jumping	3.372	5.000

Table S7. Total accuracy of motion recognition without and with using postsynaptic current change (ΔPSC) as the additional classification criteria.

**Task Name**	**Recognition Accuracy without using ΔPSC**	**Recognition Accuracy with using ΔPSC**
**Human activity recognition**	*0.85*	*0.94*
**Drone flight mode recognition**	*0.52*	*0.95*

Table S8. Performance comparison between our system and other human-motion perception systems reported elsewhere in terms of recognition accuracy and system design for motion detection and activity monitoring tasks. IMU: inertial measurement unit; CPU: central processing unit; GPU: graphics processing unit; MCU: microcontroller unit; FFT: fast Fourier transform.

**Type of human activity recognition system**	**Overall recognition accuracy**	**Hardware implementation**	**Recognition principle**	**Computational cost**
Magnetic induction-based wireless system for human activity recognition [12]	87.0% - 98.9%	Magnetic induction sensor, CPU	Machine learning classification (Deep recurrent neural network)	High (processing unit required)
Continuous human activity recognition system [13]	92.2% - 96.0%	IMU sensor (smartwatch), GPU	Machine learning classification (Deep neural network)	High (processing unit required)
Wearable activity recognition device [14]	95.1%	IMU sensor, air-pressure sensor, MCU, CPU	Machine learning classification	High (processing unit required)
Wearable IMU sensor with human activity recognition [15]	91.8% (daily) 87.8% (fall)	IMU sensor, CPU	Signal processing (FFT) & machine learning	High (processing unit required)
Memristor-based artificial dendritic neuron [16]	96% (in simulation)	Memristor array, MCU, GPU	Spatiotemporal processing using artificial dendrite neuron (bio-plausible)	Relatively low (processing unit involved; memristor based)
Synaptic electronics enabled bioinspired spatial cognition system (this work)	94.17%	IMU sensor, synaptic transistor, MCU	Spatiotemporal recognition using artificial synaptic device (bio-plausible)	Low (synaptic transistor enabled)

Table S9. Total accuracy of motion recognition achieved by using different devices.

	**Device #1 (original)**	**Device #2**	**Device #3**
**Classification accuracy (human motion)**	*0.94*	*0.95*	*0.94*
**Classification accuracy (drone motion)**	*0.95*	*0.93*	*0.95*

Table S10. Decision boundaries of synaptic response (ΔPSC in μA) for classifying 26 alphabet letters of flag semaphore.

Letter	Decision boundaries (synaptic response, μA)	
	Low	High
A	0.0000	0.4717
B	0.4717	0.6354
C	0.6354	0.8174
D	0.8174	0.9362
E	0.9362	1.0133
F	1.0133	1.1186
G	1.1186	1.2550
H	1.2550	1.3846
I	1.3846	1.4173
J	1.4173	1.4401
K	1.4401	1.4803
L	1.4803	1.5210
M	1.5210	1.5818
N	1.5818	1.6484
O	1.6484	1.7853
P	1.7853	1.9159
Q	1.9159	1.9544
R	1.9544	2.0213
S	2.0213	2.0904
T	2.0904	2.1263
U	2.1263	2.1790
V	2.1790	2.3299
W	2.3299	2.4929
X	2.4929	2.5565
Y	2.5565	2.6633
Z	2.6633	3.0000

Table S11. Decision boundaries of synaptic response (ΔPSC in μA) for classifying 6 types of hand gestures, including shaking, circling, horizontal, vertical, vertical-horizontal, and horizontal-vertical movements.

**Hand gestures**	**Decision boundaries (synaptic response, μA)**	
	**Low**	**High**
Vertical	0.000	0.270
Horizontal	0.270	0.515
Horizontal-vertical	0.515	0.737
Vertical-horizontal	0.737	1.006
Circling	1.006	2.185
Shaking	2.185	4.000

Table S12. Decision boundaries of synaptic response (ΔPSC in μA) for recognizing drone flight modes, including wind stabilization, collision stabilization, spinning, ascending (or descending), and spin-ascending (or spin-descending).

Drone flight mode	Decision boundaries (synaptic response, μA)
Wind stabilization	0.000 ~ 0.645
Collision stabilization	0.645 ~ 1.219
Spinning	1.219 ~ 1.506
Ascending	1.506 ~ 2.143
Spin-ascending	2.143 ~ 4.000

Table S13. Performance comparison between our synaptic-electronics enabled system and other sensor-processor systems reported elsewhere in terms of detection and recognition of drone motion towards robotic applications. UAV: unmanned aerial vehicle; IMU: inertial measurement unit; ARM: advanced RISC machines; MCU: microcontroller unit.

**Type of UAV motion sensing system**	**Hardware implementation**	**Algorithm and principle**	**Functions and Applications**
Small quadrotor UAV with autonomous navigation [17]	IMU, camera, flight microcontroller (ARM)	Feature extraction & matching algorithm	Pose estimation, hazard-avoiding landing
Autonomous UAV navigation system [18]	Waypoint generator, motion planner, state estimation, flight controller	Motion planning algorithm	Path planning, obstacle avoidance
Multi-robot UAV System [19]	Camera, IMU, tracker, controller	Control and estimation algorithms	Motion planning, automatic stabilization, swarm formation
Movement detector neuron [20]	Dynamic vision sensor, neuromorphic processor	Spiking neural network	Obstacle avoidance
Synaptic electronics enabled bioinspired spatial cognition system (this work)	IMU, spike-coding circuit (MCU), synaptic transistor	Spatiotemporal recognition using synaptic device (bio-plausible)	Flight mode recognition, collision recognition

Table S14. Calculation of the multisensory enhancement index (MEI) for human motion recognition task and drone flight mode recognition task. Recognition accuracy under unimodal conditions (i.e., using either gyroscope or accelerometer) and bimodal conditions (i.e., using both gyroscope and accelerometer) were computed, and then the multisensory enhancement index were derived.

Task Name	Modality 1 performance (gyroscope)	Modality 2 performance (accelerometer)	Bimodal performance	Multisensory enhancement index
Human motion recognition	0.816	0.783	0.942	15.4%
Drone flight mode recognition	0.760	0.680	0.947	24.6%

Table S15. Motion recognition performance under unimodal vestibular, unimodal visual, bimodal vestibular-visual conditions.

Task Name	Uni-modal accuracy (vestibular)	Uni-modal accuracy (visual)	Bimodal accuracy (vestibular+visual)	Multisensory Enhancement Index
Human motion recognition	*0.825*	*0.708*	*0.958*	*16.12 %*

Table S16. Motion recognition performance under unimodal vestibular, unimodal tactile, bimodal vestibular-tactile conditions.

Task Name	Uni-modal accuracy (vestibular)	Uni-modal accuracy (tactile)	Bimodal accuracy (vestibular+tactile)	Multisensory Enhancement Index
Human motion recognition	*0.825*	*0.858*	*0.950*	*10.68 %*

Supporting References:

1. Choi, H. H., Cho, K., Frisbie, C. D., Sirringhaus, H. & Podzorov, V. Critical assessment of charge mobility extraction in FETs. *Nature Materials*, **17**, 2-7 (2018).
2. Bessonov, A. A., Kirikova, M. N., Petukhov, D. I., Allen, M., Ryhänen, T. & Bailey, M. J. Layered memristive and memcapacitive switches for printable electronics. *Nature Materials* **14**, 199-204 (2015).
3. Wang, J., Teng, C., Zhang, Z., Chen, W., Tan, J., Pan, Y., Zhang, R., Zhou, H., Ding, B. & Cheng, H.-M. A Scalable Artificial Neuron Based on Ultrathin Two-Dimensional Titanium Oxide. *ACS Nano* **15**, 15123-15131 (2021).
4. Nguyen, D. A., Park, D. Y., Duong, N. T., Lee, K. N., Im, H., Yang, H. & Jeong, M. S. Large-Area MoS_2 via Colloidal Nanosheet Ink for Integrated Memtransistor. *Small Methods* **5**, 2100558 (2021).
5. Yan, X., Qin, C., Lu, C., Zhao, J., Zhao, R., Ren, D., Zhou, Z., Wang, H., Wang, J. & Zhang, L. Robust $Ag/ZrO_2/WS_2/Pt$ memristor for neuromorphic computing. *ACS Applied materials & interfaces* **11**, 48029-48038 (2019).
6. Yan, X., Zhao, Q., Chen, A. P., Zhao, J., Zhou, Z., Wang, J., Wang, H., Zhang, L., Li, X. & Xiao, Z. Vacancy-induced synaptic behavior in 2D WS_2 nanosheet–based memristor for low-power neuromorphic computing. *Small* **15**, 1901423 (2019).
7. Indiveri, G., Linares-Barranco, B., Hamilton, T. J., Schaik, A. v., Etienne-Cummings, R., Delbruck, T., Liu, S.-C., Dudek, P., Häfliger, P. & Renaud, S. Neuromorphic silicon neuron circuits. *Frontiers in neuroscience* **5**, 73 (2011).
8. Bartolozzi, C. & Indiveri, G. Synaptic dynamics in analog VLSI. *Neural computation* **19**, 2581-2603 (2007).
9. Frenkel, C., Lefebvre, M., Legat, J.-D. & Bol, D. A 0.086-mm^2 12.7-pJ/SOP 64k-synapse 256-neuron online-learning digital spiking neuromorphic processor in 28-nm CMOS. *IEEE transactions on biomedical circuits and systems* **13**, 145-158 (2018).
10. Huayaney, F. L. M., Nease, S. & Chicca, E. Learning in silicon beyond STDP: a neuromorphic implementation of multi-factor synaptic plasticity with calcium-based dynamics. *IEEE Transactions on Circuits and Systems I: Regular Papers* **63**, 2189-2199 (2016).
11. Kim, S., Kim, S., Ho, D. H., Roe, D. G., Choi, Y. J., Kim, M. J., Kim, U. J., Le, M. L., Kim, J. & Kim, S. H. Neurorobotic approaches to emulate human motor control with the integration of artificial synapse. *Science Advances* **8**, eabo3326 (2022).
12. Golestani, N. & Moghaddam, M. Human activity recognition using magnetic induction-based motion signals and deep recurrent neural networks. *Nature Communications* **11,** 1551 (2020).
13. Ashry, S., Ogawa, T. & Gomaa, W. CHARM-deep: Continuous human activity recognition model based on deep neural network using IMU sensors of smartwatch. *IEEE Sensors Journal* **20,** 8757-8770 (2020).
14. Yang, D., Huang, J., Tu, X., Ding, G., Shen, T. & Xiao, X. A wearable activity recognition device using air-pressure and IMU sensors. *IEEE Access* **7,** 6611-6621 (2018).
15. Zhuang, W., Chen, Y., Su, J., Wang, B. & Gao, C. Design of human activity recognition algorithms based on a single wearable IMU sensor. *International Journal of Sensor Networks* **30,** 193-206 (2019).
16. Li, X., Zhong, Y., Chen, H., Tang, J., Zheng, X., Sun, W., Li, Y., Wu, D., Gao, B. & Hu, X. Memristors-

based Dendritic Neuron for High-Efficiency Spatial-Temporal Information Processing. *Advanced Materials* 2203684 (2022).
17. Brockers, R., Hummenberger, M., Weiss, S. & Matthies, L. Towards autonomous navigation of miniature UAV. *Proceedings of the IEEE Conference on Computer Vision and Pattern Recognition Workshops* 631-637 (2014).
18. González-Sieira, A., Cores, D., Mucientes, M. & Bugarín, A. Autonomous navigation for UAVs managing motion and sensing uncertainty. *Robotics and Autonomous Systems* **126,** 103455 (2020).
19. Baca, T., Petrlik, M., Vrba, M., Spurny, V., Penicka, R., Hert, D. & Saska, M. The MRS UAV system: Pushing the frontiers of reproducible research, real-world deployment, and education with autonomous unmanned aerial vehicles. *Journal of Intelligent & Robotic Systems* **102,** 1-28 (2021).
20. Salt, L., Indiveri, G. & Sandamirskaya, Y. Obstacle avoidance with LGMD neuron: towards a neuromorphic UAV implementation. *IEEE International Symposium on Circuits and Systems (ISCAS)* 1-4 (2017).
